# Model uncertainty and simulated multispecies fisheries management advice in the Baltic Sea

**DOI:** 10.1371/journal.pone.0211320

**Published:** 2019-01-28

**Authors:** Barbara Bauer, Jan Horbowy, Mika Rahikainen, Nataliia Kulatska, Bärbel Müller-Karulis, Maciej T. Tomczak, Valerio Bartolino

**Affiliations:** 1 Baltic Sea Centre, Stockholm University, Stockholm, Sweden; 2 German Centre for Integrative Biodiversity Research (iDiv) Halle-Jena-Leipzig, Germany; 3 Department of Fisheries Resources, National Marine Fisheries Research Institute, Gdynia, Poland; 4 Ecosystems and Environment Research Programme, University of Helsinki, Helsinki, Finland; 5 Natural Resources Institute Finland, Helsinki, Finland; 6 Department of Aquatic Resources, Swedish University of Agricultural Sciences, Lysekil, Sweden; University of Pittsburgh, UNITED STATES

## Abstract

Different ecosystem models often provide contrasting predictions (model uncertainty), which is perceived to be a major challenge impeding their use to support ecosystem-based fisheries management (EBFM). The focus of this manuscript is to examine the extent of model disagreements which could impact management advice for EBFM in the central Baltic Sea. We compare how much three models (EwE, Gadget and a multispecies stock production model) differ in 1) their estimates of fishing mortality rates (Fs) satisfying alternative hypothetical management scenario objectives and 2) the outcomes of those scenarios in terms of performance indicators (spawning stock biomasses, catches, profits). Uncertainty in future environmental conditions affecting fish was taken into account by considering two seal population growth scenarios and two nutrient load scenarios. Differences in the development of the stocks, yields and profits exist among the models but the general patterns are also sufficiently similar to appear promising in the context of strategic fishery advice. Thus, we suggest that disagreements among the ecosystem models will not impede their use for providing strategic advice on how to reach management objectives that go beyond the traditional maximum yield targets and for informing on the potential consequences of pursuing such objectives. This is especially true for scenarios aiming at exploiting forage fish sprat and herring, for which the agreement was the largest among our models. However, the quantitative response to altering fishing pressure differed among models. This was due to the diverse environmental covariates and the different number of trophic relationships and their functional forms considered in the models. This suggests that ecosystem models can be used to provide quantitative advice only after more targeted research is conducted to gain a deeper understanding into the relationship between trophic links and fish population dynamics in the Baltic Sea.

## Introduction

There has been an increasing interest in ecosystem-based fisheries management (EBFM) in recent years. EBFM is defined as a set of principles for managing fisheries as parts of complex socio-ecological systems [1,2]. It is recognized within the scientific community that ecosystem models, which describe a broader ecological context than single-species models, have a potential role to support EBFM [3–12]. Several suggestions on the operational use of ecosystem models in fisheries management have been made. For example, to test environmental harvest control rules under a range of environmental conditions [13], to contribute to Integrated Ecosystem Assessments by developing performance metrics in line with management objectives [14,15], to identify tipping points [16] and to provide input for decision support tools, such as quantitative estimates of the expected costs and benefits of alternative management actions [8,13,17]. Ecosystem models have already been used for management in some cases, e.g. for the evaluation of the North Sea multi-annual management plan for demersal stocks ([18], see also [5,19] for other examples).

Fisheries-focused ecosystem models are simplifications of very complex socio-ecological systems. Many types of modelling approaches (e.g. statistical multispecies models, process-based food web models) emphasize diverse ecological characteristics of the system and populations (biomass, size distribution, age distribution) and vary in the level of detail in the representation of the socio-economic elements [20]. Thus, the models often include different mathematical formulations, assumptions and a varying number of components [8]. The sensitivity of model projections to the modelling approach used (model uncertainty) has been acknowledged in ecosystem modeling for decades [8,21,22]. However, the methodology to tackle model uncertainty in association with management advice is not as established as the methods for other sources of uncertainty such as sampling error, natural variability, parameter uncertainty and varying functional formulations or model resolution within a particular modelling approach [23–30]. In this study we focus on quantifying discrepancies in model outputs of hypothetical medium- term management scenario simulations and on deliberating on their impact on management conclusions.

We use simulations of the major commercial fisheries in the Baltic Sea to evaluate model agreement on management advice among three modelling approaches: Ecopath with Ecosim (EwE, [6,31]), Globally applicable Area-Disaggregated General Ecosystem Toolbox (Gadget, [32,33]) and a multispecies stock production model (MSPM, [34,35]). They represent a sample of a broad array of approaches listed by [20,36] to be potentially useful for EBFM for evaluating consequences of management actions and understanding ecosystem dynamics. They differ in modeling method (process-based vs. statistical), number of included components (few species to whole-ecosystem), detail in representing internal population structure (adults and juveniles to fully age-length structured) and in temporal resolution (annual to seasonal). The advantages and disadvantages of the approaches are described by [20,36,37]. Evaluating model uncertainty is the attempt to understand the role of model structure in the outcome of model projections. It is an important emerging need in the application of ecosystem models for EBFM [38,39] and is necessary to maximise the complementary strengths of different approaches to answer specific management questions on complex systems. Comparison of outputs produced by conceptually different models such as those applied in our study represents a challenge. For this purpose model agreement is evaluated using two indices measuring the deviation among 1) qualitative and 2) quantitative model outputs.

We argue that under an EBFM context, models could be used for strategic management advice in at least two different ways. First, models can provide insight on the level of fishing pressure required to achieve certain management goals and in the case of ecosystem models this can expand to multiple species and goals beyond maximum yields (i.e., ecosystem functions and socio-economic objectives, [3,12,40]).

Second, models may aid strategic planning by highlighting the expected long- or medium- term consequences of alternative management strategies on a large number of ecosystem features that may go beyond the traditional fishery management metrics such as fishing mortality and biomass of target fish stocks. There are a few previous studies focused on comparing the consistency of structurally different models in ranking different management scenarios. For example, [41] found that model predictions were more consistent on the consequences of some management scenarios than others which is interpreted as a measure of how robust different strategies are to model uncertainty. [42] simulated a set of scenarios involving the depletion of certain fish groups from their unfished biomasses using two ecosystem models and compared the predicted biomass responses of other functional groups in the food web. Similarly, [43] compared the ranking of management scenarios by two ecosystem models. Scenarios in their study were defined as the fishing efforts achieving economic or ecological objectives and ranked according to catch-based and biomass-based indicators. Both studies highlighted the importance of assumed trophic structure of the ecosystem on general model behavior.

We examined the impact of model uncertainty on the management advice (as described above) using five management scenarios, corresponding to five alternative management strategies. The strategies differed in their objectives including economic and conservation objectives. Three of the strategies tested included maximizing the profit of different fisheries compartments (i.e., pelagic versus demersal fisheries) and one of the strategies aimed at the recovery of a depleted predatory fish population. Subsequently, we investigated the medium-term performances of the alternative management scenarios in terms of indicators describing relative changes in spawning stock biomass (SSB), catch and profit compared to current levels. The design of fisheries management alternatives and the selection of indicators that describe to which extent the objectives had been achieved was carried out iteratively with stakeholders (e.g. managers, industry representatives) from Baltic Sea countries to ensure the relevance of the tested management alternatives and indicators. We recognize that changing environmental factors may modify the outcomes of fisheries management scenarios [5]. Thus, we simulate the alternative fisheries management scenarios across a few distinct environmental scenarios.

To summarise, we compare ecosystem model outputs from several perspectives. First, we investigate how the F-yield relationship varies across the models. Second, we compare the multispecies Fs that maximize the objectives of alternative fishery management strategies according to each model…. Third, we quantify model agreement on the simulated outcomes of those alternative strategies and examine the reasons for disagreements among models.

## Methods

### Description of the study system

The central Baltic Sea pelagic fish biomass is dominated by two clupeid stocks, Baltic sprat (*Sprattus sprattus*) and central Baltic herring (*Clupea harengus*). Both are important consumers in the pelagic food web [44], serve as key food for the Eastern Baltic cod stock (*Gadus morhua*, [45,46]) and form part of the diet of seals as well [47]. They compete for food [48], and herring growth is negatively affected by high sprat densities [49]. Changes in the salinity, temperature and oxygen concentration affect food availability, recruitment conditions and growth of cod, herring and sprat [50–52].

Cod, herring and sprat represent the main target species of the Baltic Sea harvest fisheries, making about 95% of the total catches [53]. Demersal trawlers and gillnetters mainly target cod, while pelagic trawlers target herring and sprat [53].The cod, herring and sprat fisheries in the Baltic are governed by the EU as guided by the Common Fisheries Policy (CFP). Annual total allowable catches (TAC) are set on the basis of a multiannual management plan which is based on ranges of F levels that provide yields not less than 95% of MSY [54] F ranges are used in an attempt to consider ecosystem consequences of the fishery and management, e.g. a stock can be fished at higher F than F_MSY_ (but below the upper F range) if it is necessary to avoid serious harm to the stock caused by intra- or interspecific stock dynamics.

### Ecosystem models

**Fig 1:**
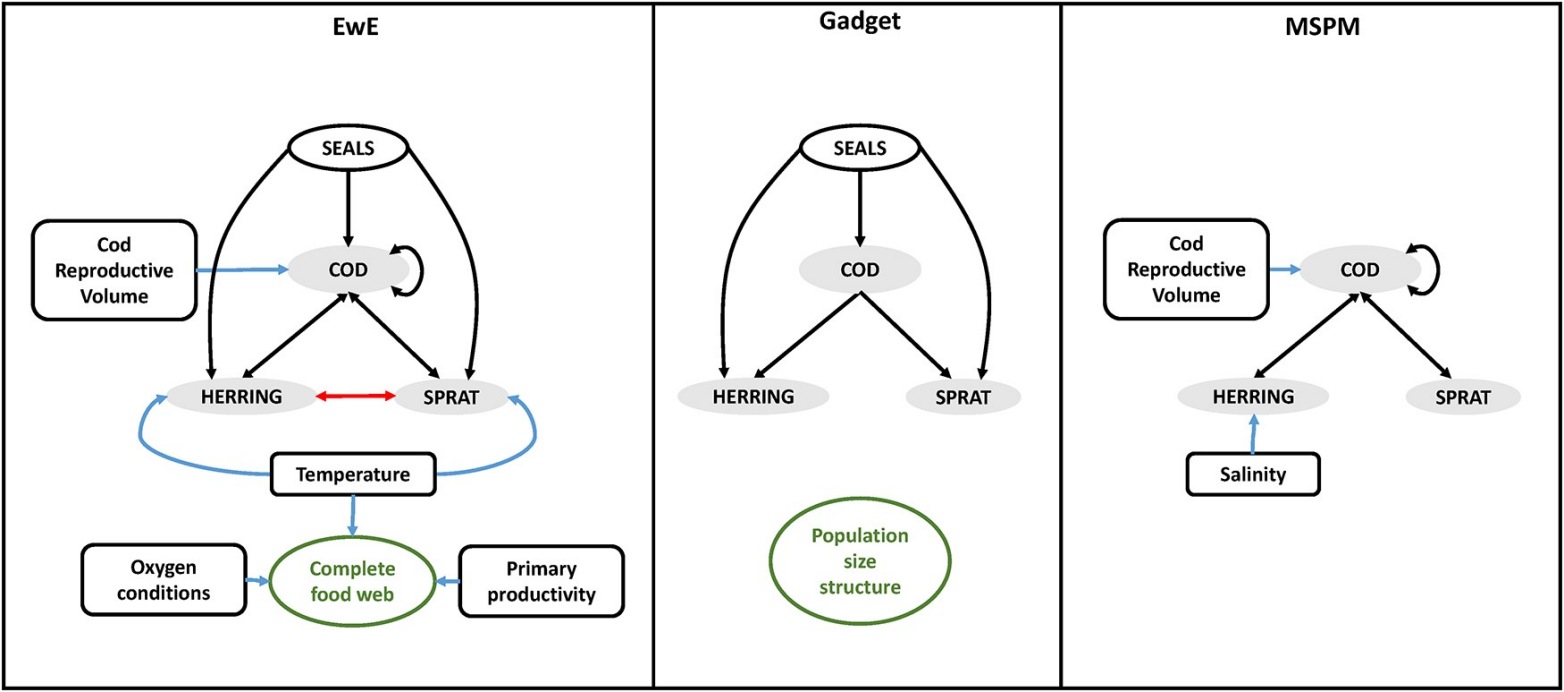
Model components and interactions in (A) EwE, (B) Gadget and (C) MSPM models of the Baltic Sea ecosystem. Grey shaded ovals represent the three fish stocks included in all models. Green ovals denote components unique to a model. Black arrows represent predation, the red arrow competition, and blue arrows abiotic environmental effects. Single-headed arrows represent one-way interactions and double-headed arrows dynamic predator-prey feedbacks. Fitting periods for the models were 2004–2013 (EwE), 1974–2013 (Gadget) and 1982–2013 (MSPM). EwE and MSPM have yearly, while Gadget quarterly time steps.

Major components and interactions represented in the three ecosystem models we used are shown on Fig 1. A more detailed description of the modelling approaches and their implementation for the Baltic Sea can be found in the S1 Appendix, chapters 1–4.

**Ecopath with Ecosim** (EwE; [6,31] is a commonly used software and process-based modelling approach to study whole-ecosystem effects of fisheries. The EwE model of the open Baltic Sea [55] includes charismatic species such as grey seals and offshore fish-feeding birds, four fish species (cod, herring, sprat and flounder), the benthic part of the food web, four zooplankton groups and one phytoplankton group. The Ecopath component represents biomass flows among organismal groups within the food web and to fisheries in the ‘model year’, 2004. Ecosim dynamically simulates the temporal development of biomasses and catches in the system 2004–2013 given certain fishing mortalities (defined as yearly harvest rate, catch/biomass) and environmental forcing. EwE model simulations have been carried out using the software Ecopath with Ecosim v. 6.5.

**Gadget** is a platform to run statistical models of marine ecosystems consisting of a limited number of species, accounting for biological processes, such as maturation, growth, predation, etc. [56,57]. The Gadget implementation in the Baltic is a multispecies and multifleet model. Trophic interactions are represented by cod feeding on both herring and sprat, as well as on benthic prey. The model is age-length structured with quarterly time steps running from 1974 to 2013. The current implementation uses the package Rgadget v.0.5 [58] under R v.3.4.1 [59].

**The MultiSpecies Production Model (MSPM,** [34,35] is a simplification of the age-structured multispecies model of [60]. The model was applied to simulate stock dynamics in yearly time steps and interactions of the cod, herring, and sprat stocks in the central Baltic from 1982 to 2013. It considers the trophic interactions among these stocks (predation of cod on herring, sprat, and young cod), the environmental impact on growth of cod and herring and density dependent growth of sprat. Predation in the model depends on the biomass of available food, thus cod cannibalism is dependent on clupeid biomass. Model simulations were run using Excel 2010 with VisualBasic.

All three models provide information about the biomasses of three stocks of interest (cod, herring, sprat) in the central Baltic (Fig 1), with EwE and MSPM providing limited information about the internal structure of each stock (both simulating only a few stanzas per stock). Only Gadget represents age and size structure of populations in both biomass and numbers. On the other hand, EwE and MSPM both incorporate the dependency of the cod biomass growth on prey availability, although partly through different mechanisms (Table 1), while in Gadget the growth of cod is not limited by the amount of available prey biomass. Both direct and indirect interactions of seals with the fisheries via predation for the same fish resources and via damage of the catches in the small-scale fisheries are accounted for by EwE and Gadget but not by MSPM. For a summary of model assumptions see Table 1.

**Table 1:** Comparison of key assumptions with respect to population structure (1–3), trophic interactions (3–6) and human and abiotic environmental pressures (7–8).

Model assumptions on	Ecopath with Ecosim	Gadget	Multispecies Stock Production Model
1. Fish population structure	Biomass density (t/km^2^) of adult and young fish components	Total number of individuals in stock; Length-age groups, biomass derived from length-weight relationship	Total biomass (t) of adult and young fish components
2. Stock-recruitment (SR) relationship	Complex, number of recruits depends on weight-dependent fecundity and on environmental forcing	Hockey-stick on all stocks	Cod: Ricker; herring and sprat: Beverton-Holt
3. Cod predation	Increases up to a maximum level with increasing cod biomass and linearly increases with prey biomass. Maximum predation mortality by cod much lower for herring than for sprat.	Increases up to a maximum level with increasing cod biomass and increases with prey availability.	Predation mortality of a certain prey linearly increases with increasing cod biomass and declines with total prey biomass
4. Prey feedback on cod	Population growth varies with food consumption	Not included	High biomass of clupeids leads to decline in cod cannibalism and vice versa
5. Clupeid predation	Dynamics explicitly modelled, clupeids compete for shared prey	Not included	Not included
6. Seal predation	Saturating function of seal biomass and linear function of prey biomass. Potential increase of predation mortality by seals on any prey compared to current levels is limited	Linear function of seal biomass and increasing function of prey biomass	Not included
7. Abiotic environmental dependency	Reproduction, consumption rates of zooplankton and some benthos groups, primary production	Not included	Growth efficiency (anabolism rate, only cod and herring)
8. Fishing mortality (F) implemented as	Annual catch/biomass	Quarterly catch/harvestable biomass in respective quarter	Instantaneous fishing mortality

To focus on structural differences and minimize data-driven differences among models, we used the same datasets for model parametrization (e.g. surveys, commercial catches and assessments, see Table D in S1 Appendix, chapter 4) as far as it was possible. A common database containing cod stomach survey data was used to parametrise the diet of cod, the most important predator in all models [61]. We tested hindcast performance of the models by comparing simulated values to observations (catch, profit) or to the mean value of the three model estimates (biomass, demersal to pelagic ratio) from 2004–2012, which is the cross-section of the calibration periods of the models.

### Performance indicators

We considered three indicators: spawning stock biomass (SSB), catches (by stocks) and profit (by fleet segments). In EwE and MSPM SSB was approximated as the biomass of the adult fish components. In Gadget SSB was estimated using age specific proportion of mature fish [62] and results from quarterly time steps were aggregated into yearly means before any further analysis. There are three fleet segments of interest defined in the case study: 1) active (bottom trawls, BT) and 2) passive (gillnets, longlines, mostly small-scale fishery, GN) gears targeting cod and 3) pelagic trawl (PT) fishery targeting sprat and herring.

In EwE and MSPM the amounts of cod landings by BT vs. GN are calculated based on the total amount of cod catch in the model output (based on F). Total cod catches were distributed to the two fleet segments based on the relative amount of cod catches in 2013 (landings data from ICES), which were 83% BT and 17% GN. Gadget predicts cod catches separately for BT and GN assuming that their relative contribution to the catch follows the average of their relative harvest rate in the period 2009–2013 and their specific selection patterns. We assume all catches as landings since we expect discards to be reduced in the future because of the implementation of the landings obligation within the European Union.

In all models, the profit in each year is calculated as landings multiplied by price minus costs. Fish prices are based on Swedish sale notes (average values 2011–2015). In the case of cod, costs are calculated as F times a cost coefficient [63,64]. Cost coefficient parameters are calculated separately for BT and GN fisheries based on the data by the Scientific, Technical and Economic Committee for Fisheries (STECF). In the Gadget and EwE models profits in the cod fishery are negatively affected by seal abundance. We assume that increasing seal abundance increases the amount of damaged and therefore discarded cod within the passive gears segment. Costs of the clupeid fisheries are calculated as landings multiplied by a cost coefficient. Cost coefficient parameters are taken from [65]. Profit of PT is calculated as the sum of herring and sprat profits. More details on the calculation of costs, profits and the effect of seal abundance on profits are included in S1 Appendix, chapter 6.

### Model simulations

**Fig 2:**
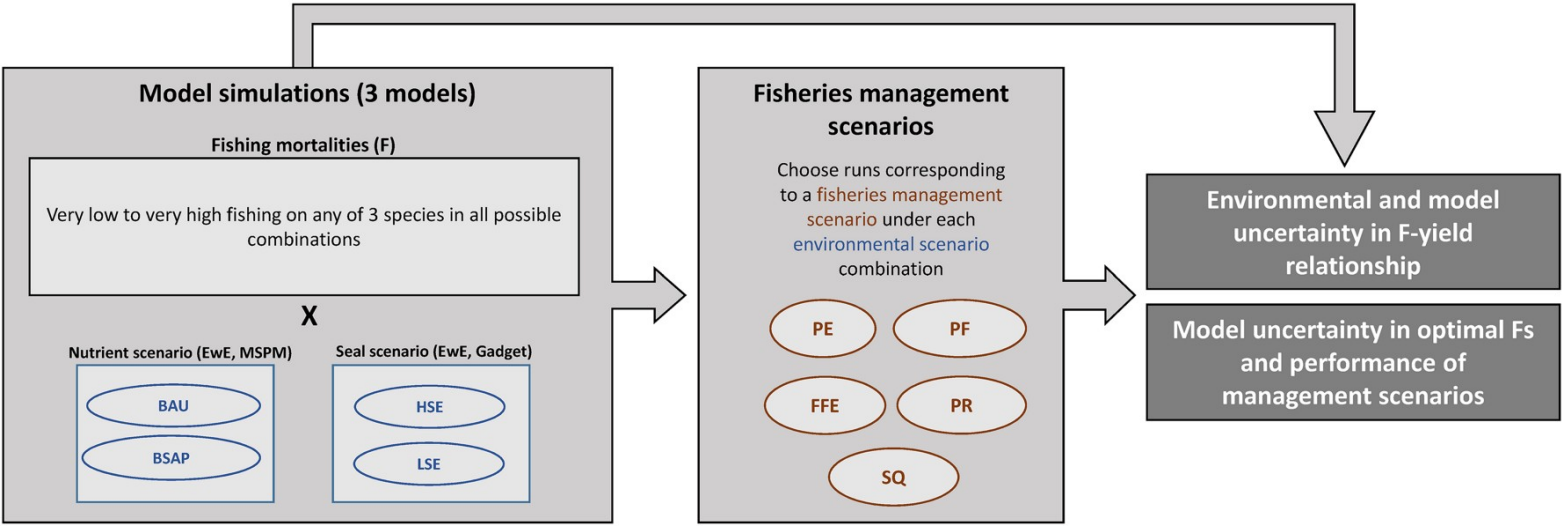
Summary of the framework. Light shaded rectangles describe the main steps of the modelling work, dark shaded rectangles indicate scientific products potentially useful for ecosystem-based fisheries management. See Table 2 for description of alternative fisheries management scenarios and Methods for the definition of nutrient and seal scenarios.

We used a multi-factorial simulation design: one simulation was run for each unique combination of two nutrient load scenarios (‘Business-As-Usual’, BAU, and Baltic Sea Action Plan, ‘BSAP’) and/or two seal population growth scenarios (Low Seal, LSE, and High Seal, HSE), and fishing mortality (varied in a range, e.g. 0–1.4, among simulations), for the years 2014–2032 (Fig 2). This resulted in a large number of simulations, and we calculated temporally aggregated metrics, such as cumulative yield and profit during the whole modelled period for meaningful comparison of the outcomes. We investigated the F-cumulative yield (= summed catches 2014–2032) relationship under each environmental scenario based on these simulations.

**Table 2:** Alternative management scenarios.

Scenario	Objective^a^	
Piscivore Exploitation (PE)	Maximize summed cumulative discounted profit of bottom trawlers (BT) and gillnetters (GN) based on their cod catches	$max\sum_{y=2014}^{y=2032}P_{c}$
Forage Fish Exploitation (FE)	Maximize cumulative discounted profit of pelagic trawlers (PT) based on their herring and sprat catches	$max\sum_{y=2014}^{y=2032}{(P}_{h}+P_{s})$
Portfolio Fishery (PF)	Maximize cumulative discounted total fisheries profits.	$max\sum_{y=2014}^{y=2032}P_{c,h,s}$
Piscivore Recovery (PR)	Maximize cod biomass compared to clupeids with constraint keeping herring and sprat at viable levels^b^	$\text{max}(\;{\overset{-}{\frac{B_{c}}{B_{s}+B_{h}})}}_{2028}^{2032},$ ${\overset{-}{{SSB}_{h}}}_{2028}^{2032}>B_{lim,h}$ ${\overset{-}{{SSB}_{s}}}_{2028}^{2032}>B_{lim,s}$
Status Quo (SQ)	Fs set to average of the last three years’ (2011–2013) values in model hindcast	

^a^Fishing mortality rate (F) values were individually selected in each model according to the objectives of each scenario, except for the ‘Status Quo’ scenario which represents a continuation of current practices. *P*_*i*_ values represent yearly discounted profits to net present value from fisheries on stock *i*, *B*_*i*_ annual biomasses and *SSB*_*i*_ spawning stock biomasses of stock *i*, where the subscripts *c*, *h* and *s* refer to cod, herring and sprat, respectively.

^b^*B*_*lim*_ values were taken from ICES assessments [53], rescaled in each model based on the correlation of model hindcasts on SSB with ICES estimates (for details see S1 Appendix, chapter 7).

The two nutrient management scenarios [66] have an effect on the eutrophication of the basin and severity of oxygen depletion. One is BAU, assuming increasing nutrient loads in the future. The other scenario, BSAP, assumes future nutrient loads corresponding to the Baltic Sea Action Plan. Both of these scenarios were simulated assuming climate change as in the IPCC scenario A1B, implying moderate warming [67], based on regionally downscaled outputs from the HadCM3 global climate model [68]. Environmental scenarios were simulated by BALTSEM [69,70], a hydrodynamical-biogeochemical model and its output was used to force the multispecies models. Thus, environmental scenarios are realistic projections of possible future developments taking into account irregular events, such as saltwater inflows. Scenario generation using BALTSEM is described in S1 Appendix, chapter 5. BALTSEM scenarios were implemented in EwE and MSPM as described in Table E in S1 Appendix, chapter 5. The two models also used BALTSEM hindcast results for the periods 2004–2013 and 1982–2012, respectively, as forcing during model calibration. The two seal population growth scenarios investigated include 5% (LSE) and 10% (HSE) growth rates.

Subsequently, we analysed simulated model advice in five hypothetical management scenarios (Fig 2). Here, we define ‘management scenario’ by a given management key objective, for example the profit for one or more of the Baltic fisheries (Table 2). Fisheries management under the ‘Piscivore Exploitation’ and the ‘Forage Fish Exploitation’ scenarios aims to maximize profits of the cod and clupeid fisheries, respectively, and the total profits under the ‘Portfolio Fishery’ scenario. We assume that in each of these scenarios fisheries aim to maximize cumulative discounted profits, i.e. net present value by applying a 3% discount rate. In the case of the ‘Piscivore Recovery’ management scenario, the cod-clupeid biomass ratio is maximized. As this scenario represented the most ‘environmentally conscious’ management of all scenarios considered, we added the criteria that herring and sprat spawning stock biomasses (SSB) could not fall below minimum levels (*B*_*lim*_). The ‘Status Quo’ scenario was defined as the continuation of ‘current’ fishing practices, that is, fishing mortalities corresponding to the average of values 2011–2013.

From all simulations, we selected those where the management scenario objective was satisfied, separately for each nutrient and seal scenario and each model (Fig 2). For example, from all simulations of the EwE model run under the combination of the BAU nutrient and HSE seal scenario, we selected those where cod/clupeid/total profits were the highest (‘Piscivore Exploitation’/’Forage Fish Exploitation’/’Portfolio Fishery’ scenario, respectively), those that fulfilled biomass criteria (‘Piscivore Recovery’) or where Fs equaled those of the average of the last three years (‘Status Quo’). We repeated this for Gadget and MSPM, and we compared model agreement on Fs and a set of performance indicators corresponding to each of the five management scenarios, and we repeated this for all combinations of environmental scenarios. We assumed that models insensitive to nutrient or seal scenarios provide the same information in both scenarios (e.g. Gadget outputs under BAU and BSAP are equivalent).

### Model agreement

We define model agreement as being negatively related to the dispersion of model outputs (the more deviating outputs models provide, the less they agree). Model agreement was quantified in two ways. The first method (‘*A*’ index) is based on measures of dispersion of qualitative variables, e.g. diversity indices. It aims to describe model agreement regarding relative trends, including cases when a model does not provide information (i.e., according to the model, multiple possible trends are possible). The second index is based on a quantitative measure of dispersion, the coefficient of variation (CV). We calculate 1/CV, the inverse of the CV of numeric model outputs (thus, only including cases when models provide such outputs). We determine model agreement using these two methods both in terms of selected Fs and performance of management scenarios.

The ‘*A*’ index measures how often for one management scenario the different models provide the same information on Fs (‘how much does F need to be changed to reach the management objective of the scenario?’) or management scenario performance (‘how is a performance indicator going to change under that scenario?’). ‘A’ also considers the influence of environmental scenarios. For example, if one model provides different information on a performance indicator under the same management scenario, but different environmental scenarios, ‘A’ becomes lower. This way ‘A’ integrates model and environmental uncertainty of the advice. We calculate *A*_*i*_ as

$$A_{i}=\sum_{j=1}^{j=6}\frac{N_{ij}(N_{ij}-1)}{N_{i}(N_{i}-1)},$$ (1)

where *N*_*i*_ is the total number of cases when model information on *i* (selected F for a stock or a performance indicator) is provided, of which *N*_*ij*_ belong to the category *j* ($j=1,2,\ldots 6;\;\sum_{j}N_{ij}=N_{i}$). We use the following 6 categories: compared to average 2011–2013 values, *1*: more than 20% decrease, *2*: 10–20% decrease, *3*: not more than +/- 10% change, *4*: 10–20% increase, *5*: more than 20% increase, *6*: no information. For example, $N_{F_{cod}}=3*2*2=12$ when calculating $A_{F_{cod}}$ for a particular management scenario on the advice on F for cod (3 models, 2 environmental and 2 seal scenarios) and $N_{{SSB}_{cod}}=12*13=156$ when calculating $A_{{SSB}_{cod}}$ on scenario performance for the SSB of Eastern Baltic cod, as we include SSB outputs from 13 years (each year 2020–2032). We exclude the years 2014–2019 from the analysis of performance indicators (SSBs, catches and yearly profits) as transition period. To calculate *A*, the overall model agreement on one scenario, *A*_*i*_ values calculated across stocks (selected Fs) or performance indicators (scenario performance) are averaged. In Gadget, clupeid Fs have no influence on cod yields and therefore profits, and thus, nor values for clupeid Fs that maximize cod profits (the objective of the ‘Piscivore Exploitation’ scenario) neither corresponding stock status or catches of clupeids could be estimated. The category ‘no information’ was used in the case of Gadget, referring to clupeid Fs and related indicators (SSBs of herring and sprat, profit of pelagic trawls and total profit) in the ‘Piscivore Exploitation’ scenario.

## Results

### Consistency of models’ historical projections

**Fig 3:**
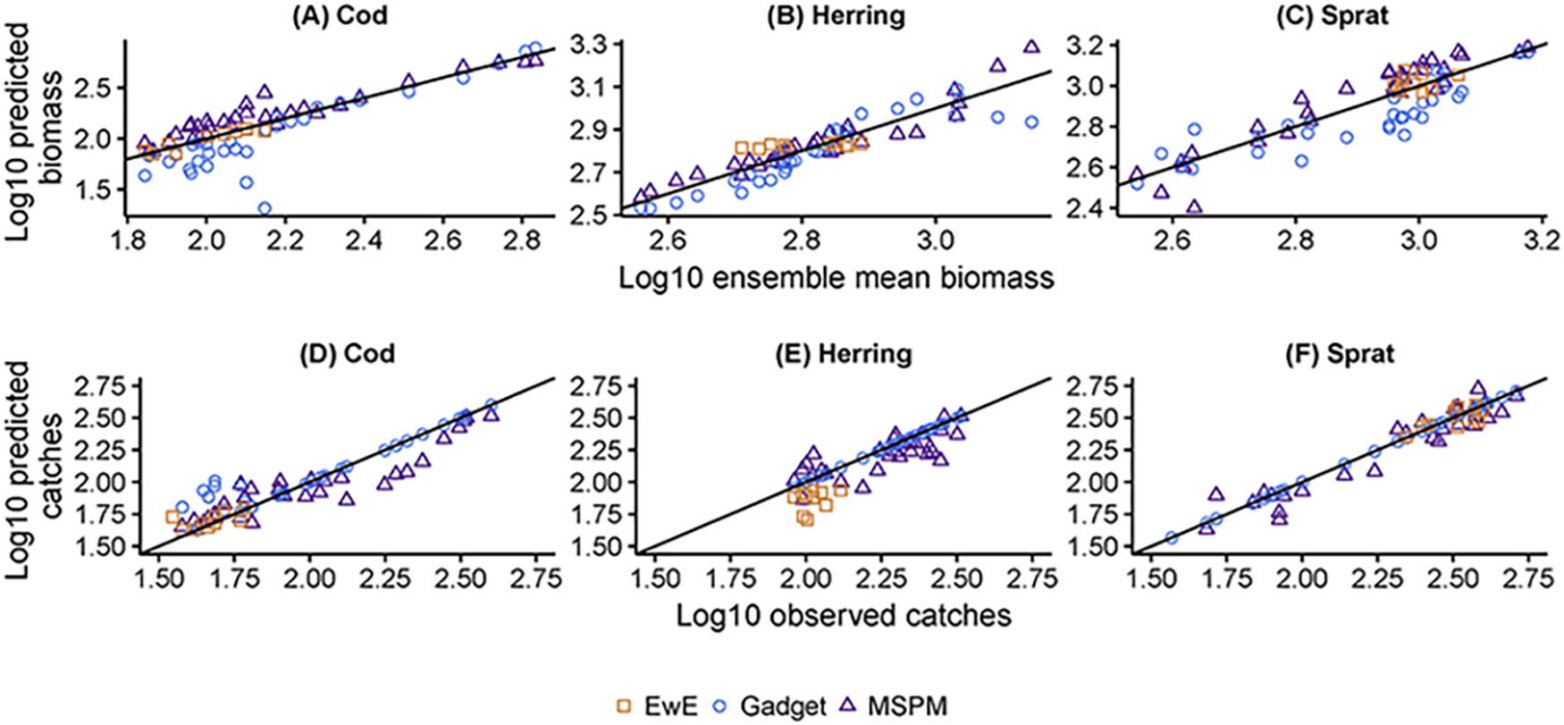
Comparison of model estimates to historical data. Model estimates of spawning stock biomass (A-C) and catches (D-F) from EwE (orange squares), Gadget (blue circles) and MSPM (purple triangles) in relation to the ensemble mean spawning stock biomass (A-C) and observed catches (D-F) of cod (A, D), herring (B, E) and sprat (C, F). The solid black line of slope = 1 and intercept = 0 represents perfect correspondence. Please note that model hindcast period was different among the models (see Methods) and therefore EwE is represented by less data points. Catches in the Gadget model are set to be equivalent to data in case of herring and sprat and for all years <2004 in the case of cod (Table D in S1 Appendix, chapter 4).

The consistency of models is tested on their historical projections with respect to adult fish biomasses and catches (Fig 3). This is done to identify potential systematic differences among models that could carry over to scenarios. The models generally agree in the trends and there are no systematic large deviations between the model output and historical catch or biomass data, except the consistent underestimation of herring catches by EwE and underestimation of cod biomass by Gadget in the lower biomass range compared to the other two models.

### Fishing mortality-yield relationships under environmental and trophic influence

**Fig 4:**
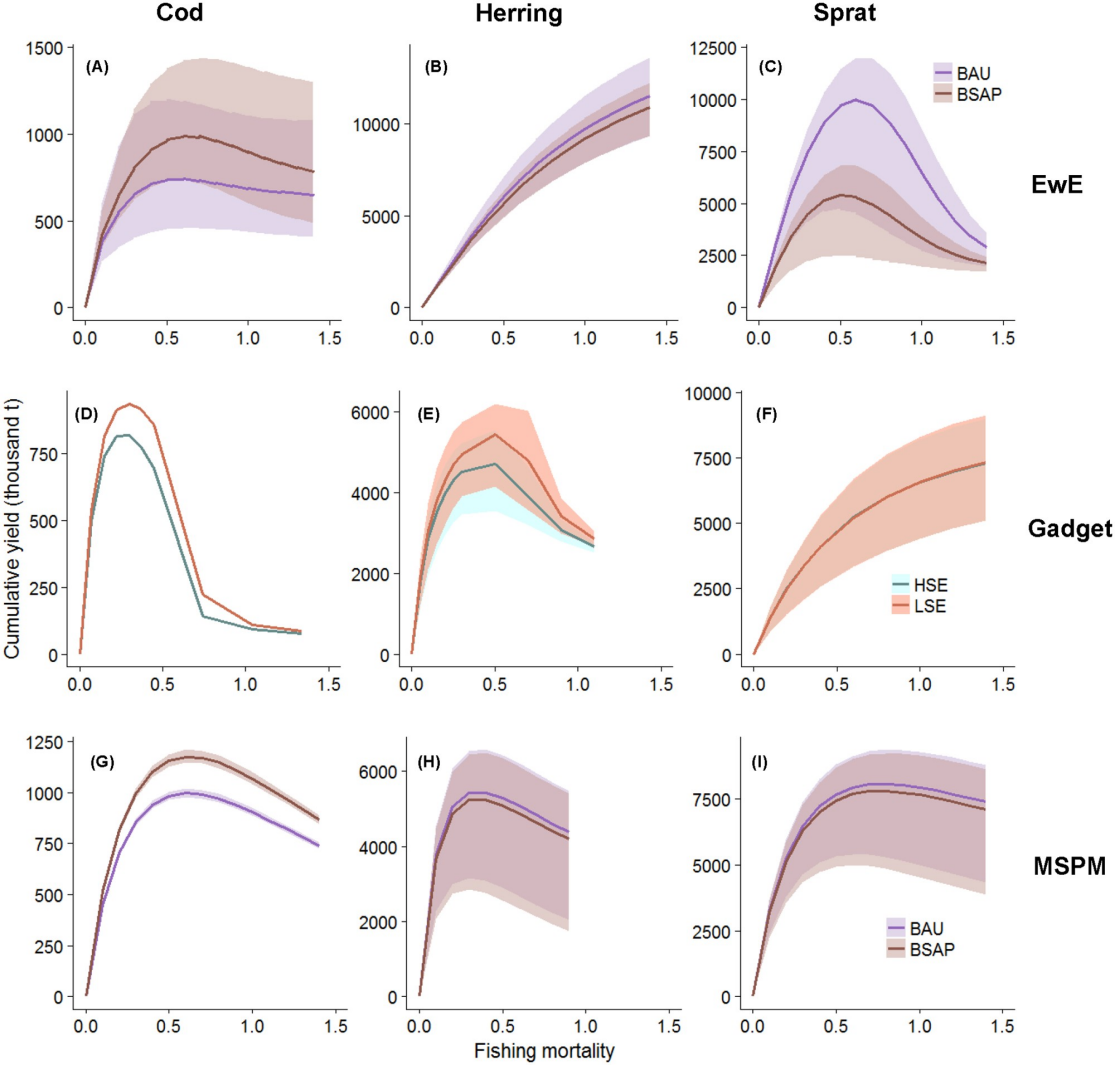
Relationship between fishing mortality rate (F, Table 2) and cumulative (2014–2032) yield for cod (left), herring (middle) and sprat (right column) projected by EwE (A-C), Gadget (D-F) and MSPM (G-I), under all cross-combinations of Fs for other stocks across all environmental scenarios. Shaded areas indicate the effect of varying the Fs of other stocks on the yield of the focal stock. Purple areas indicate the range of cumulative yields under the Business-As-Usual (BAU) and brown ones those under Baltic Sea Action Plan (BSAP) scenario (EwE, MSPM). Light blue areas indicate the range of cumulative yields under the high seal growth rate (HSE) scenario, and orange ones those under the low seal growth (LSE) scenario (seal effect visible in Gadget but not in EwE).

The relationship between F and cumulative (2014–2032) yield is saturating or dome-shaped for all stocks and all models, although herring yields in EwE and sprat yields in Gadget saturate only at unrealistically high values. In most cases there is a variation in yield at a given combination of F and environmental scenario. This is due to the fact that each of these were simulated in combination with a range of F of other stocks. In EwE such variation is high for all three stocks (Fig 4A–4C), which points to a large effect of food web interactions in that model. In the other two models, trophic interactions modify the yield of clupeids, but not that of cod, as there is no (Gadget) or only limited (MSPM) feedback from clupeid biomass to the cod dynamics (Table 1).

In both EwE and MSPM the BSAP nutrient scenario results in higher cod yield than in the BAU nutrient scenario. In Gadget, the herring and especially cod yields are negatively affected by an increase in the growth rate of the seal population, while sprat is unaffected. On the contrary, in EwE the difference between the two seal growth scenarios is negligible.

### Selected fishing mortalities (Fs)

**Fig 5:**
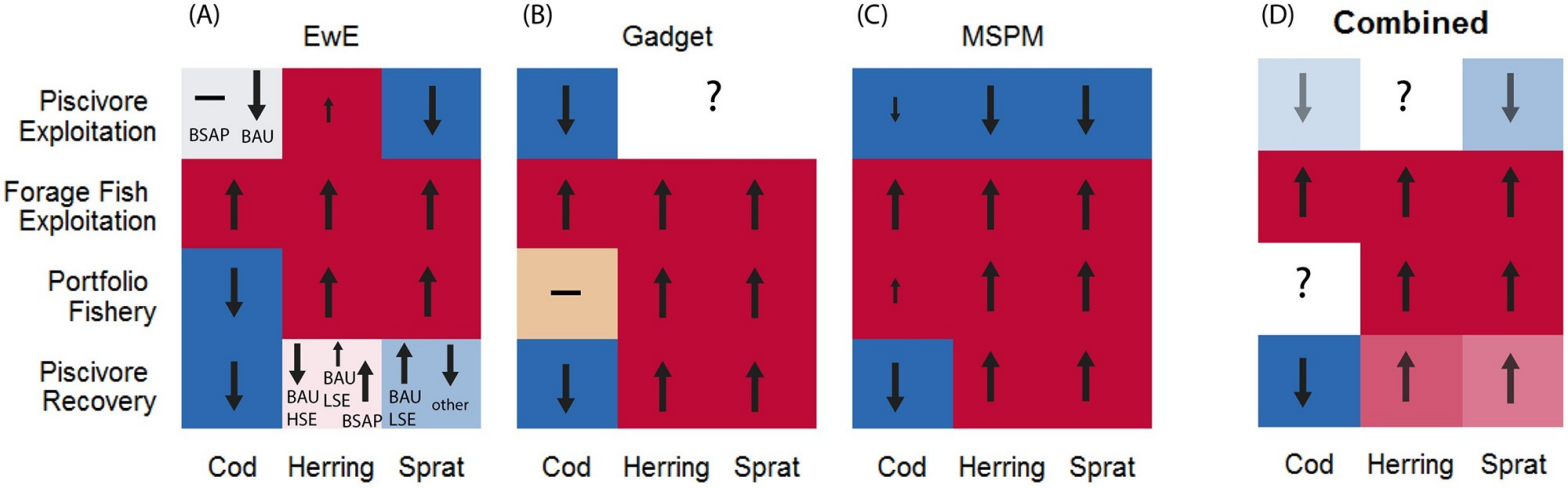
Required increases (upward arrows, red tiles), decreases (downward arrows, blue tiles) by 10–20% (small arrows) or more (large arrows), or no change (hyphen, beige tiles) compared to Status Quo Fs to achieve objectives of each management scenario according to each model (A-C) and combined (D). Lighter tiles indicate uncertainty in advice. In (A) they indicate cases when the advice provided by EwE on F was sensitive to the environmental scenario. In (D) light tiles indicate model differences in the combined information from (A)-(C). White tiles (question mark) represent cases when the model does not inform about Fs maximizing scenario objectives (B) or completely contradictory information from the model ensemble (D). BSAP indicates ‘Baltic Sea Action Plan’, BAU ‘Business-As-Usual’, HSE ‘high seal growth’ and LSE ‘low seal growth’ scenarios. ‘Other’ in (A) refers to all other scenario combinations except of BAU-LSE.

There is a mixture of agreement and disagreement among models regarding selected Fs in the different management scenarios (Fig 5). We found the largest model disagreement on the ‘Piscivore Exploitation’ scenario (Table 2), aiming to maximize profits of the cod fishery. Although all three models agreed that under this scenario cod F needs to be decreased (except in EwE under the BSAP scenario, when it stays close to current levels), they differed in their selected Fs for clupeids. EwE suggests that the maximum profit in the cod fisheries could be achieved by decreasing F on sprat, a prey of cod which is though less preferred than herring, but more vulnerable to cod predation in the model. The EwE simulation also suggests to slightly increase herring F, which is either related to the competition between herring and cod for benthic food or between herring and sprat for pelagic food. Gadget, since it does not include any feedback of prey on predator (Table 1), does not provide any information on which clupeid Fs are maximizing cod profits. In MSPM there is no competition between clupeids, so both prey F is decreased to increase both clupeids’ biomass, which gives more available food for cod and thus leads to reduction of cod cannibalism.

Under the ‘Forage Fish Exploitation’ scenario all stocks were fished at high levels in all three models. High fishing on the predator cod enabled most clupeid production to serve the fisheries. The ‘Portfolio Fishery’, a scenario aiming to maximize total fishery profits, only differs from the previous scenario in cod fishing: in EwE fishing on cod is lowered, in Gadget kept around the current level and in MSPM just slightly increased. This indicates that increasing cod Fs substantially, as in the ‘Forage Fish Exploitation’ scenario, would decrease total profits of the fishing sector overall. The ‘Piscivore Recovery’ scenario aimed at increasing the ratio of cod compared to clupeids while maintaining the latter above safe biological limits. This was generally achieved by low overall fishing levels of cod and increased fishing on clupeids. However, selected clupeid Fs were strongly dependent on environmental scenarios in EwE. Compared to the other two models the output of EwE was more sensitive to the environmental variation, as in this model relative population growth conditions of the three stocks are strongly influenced by e.g. primary productivity, hypoxia and temperature in interaction with seal predation (Fig 1, Table 1).

### Performance of fisheries management scenarios

**Fig 6:**
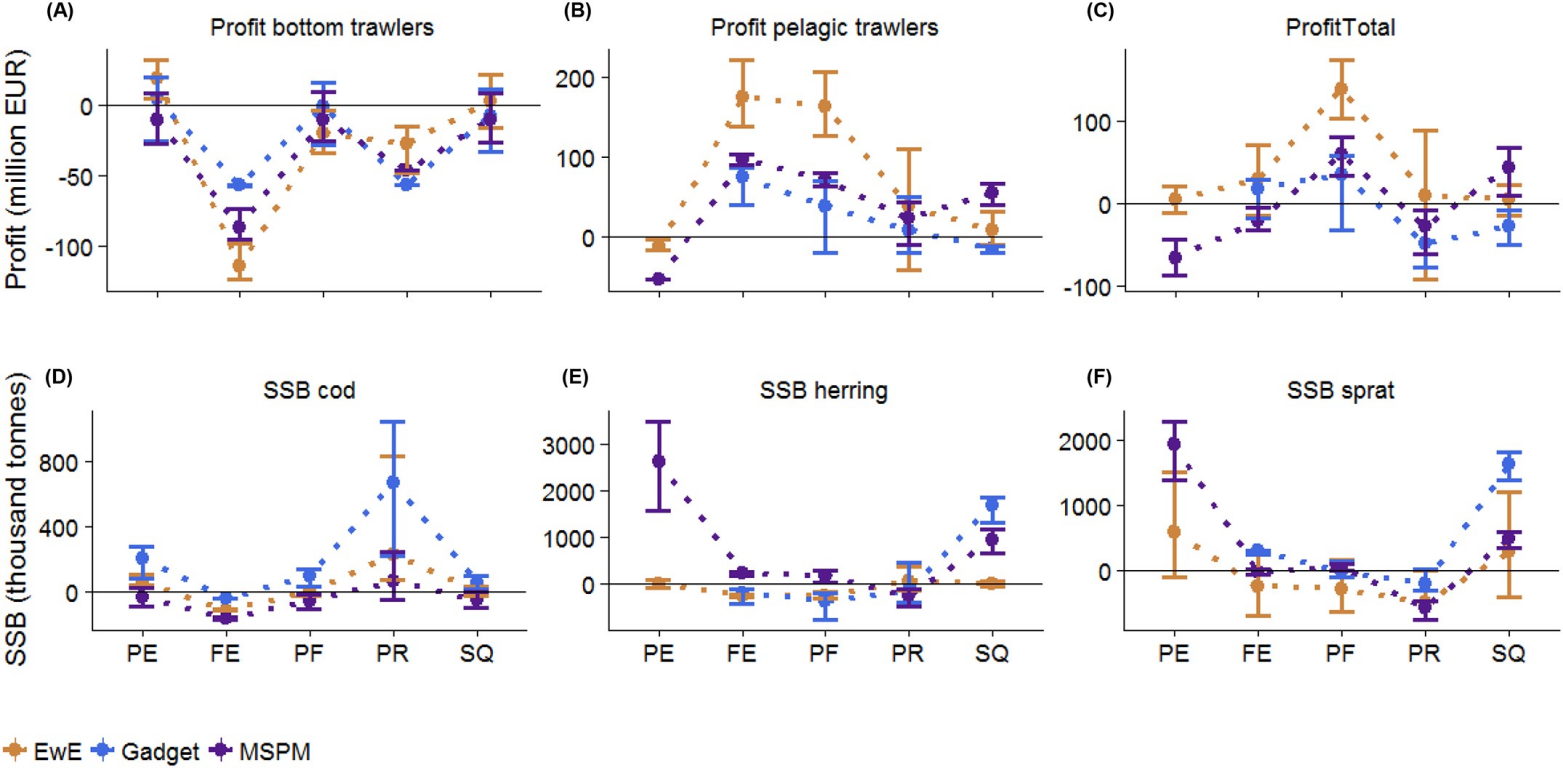
Performance indicators in fisheries management scenarios. Change in yearly profits (2020–2032) of bottom trawlers (A), pelagic trawlers (B) and of the whole fishery (C) and in the spawning stock biomass indicator of cod (D), herring (E) and sprat (F) relative to the average modelled value of 2011–2013 by EwE (brown), Gadget (blue) and MSPM (purple). Dots correspond to the mean and bars to the full range of values. Please note that Gadget does not provide information on clupeid-related indicators in the ‘Piscivore Exploitation’ (PE) scenario (see Methods).

All models projected decreasing profits for the bottom trawlers (Fig 6A) and increasing profits for pelagic trawlers (Fig 6B) in all scenarios, with a few exceptions. These trends resulted in total profits of the fishery staying around the reference (average 2011–2013) values, except the ‘Portfolio Fishery’ scenario where total profits of the fishery increased (Fig 6C). This is not surprising as in this scenario Fs were chosen to maximize total profits. Variability among model projections in terms of SSB’s was larger than for profits, as in some cases individual models predicted much higher SSB’s than the other two models (Fig 6D–6F). Nevertheless, general trends were similar among models. Cod biomass was highest in the ‘Piscivore Recovery’ scenario, when it was fished at a low level and lowest in the ‘Forage Fish Exploitation’ scenario (Fig 6D), as it was depleted to minimize its predation on herring and sprat. The ‘Status Quo’ and ‘Piscivore Exploitation’ scenarios resulted in the highest biomasses of herring (Fig 6E) and sprat (Fig 6F).

### Model agreement

**Fig 7:**
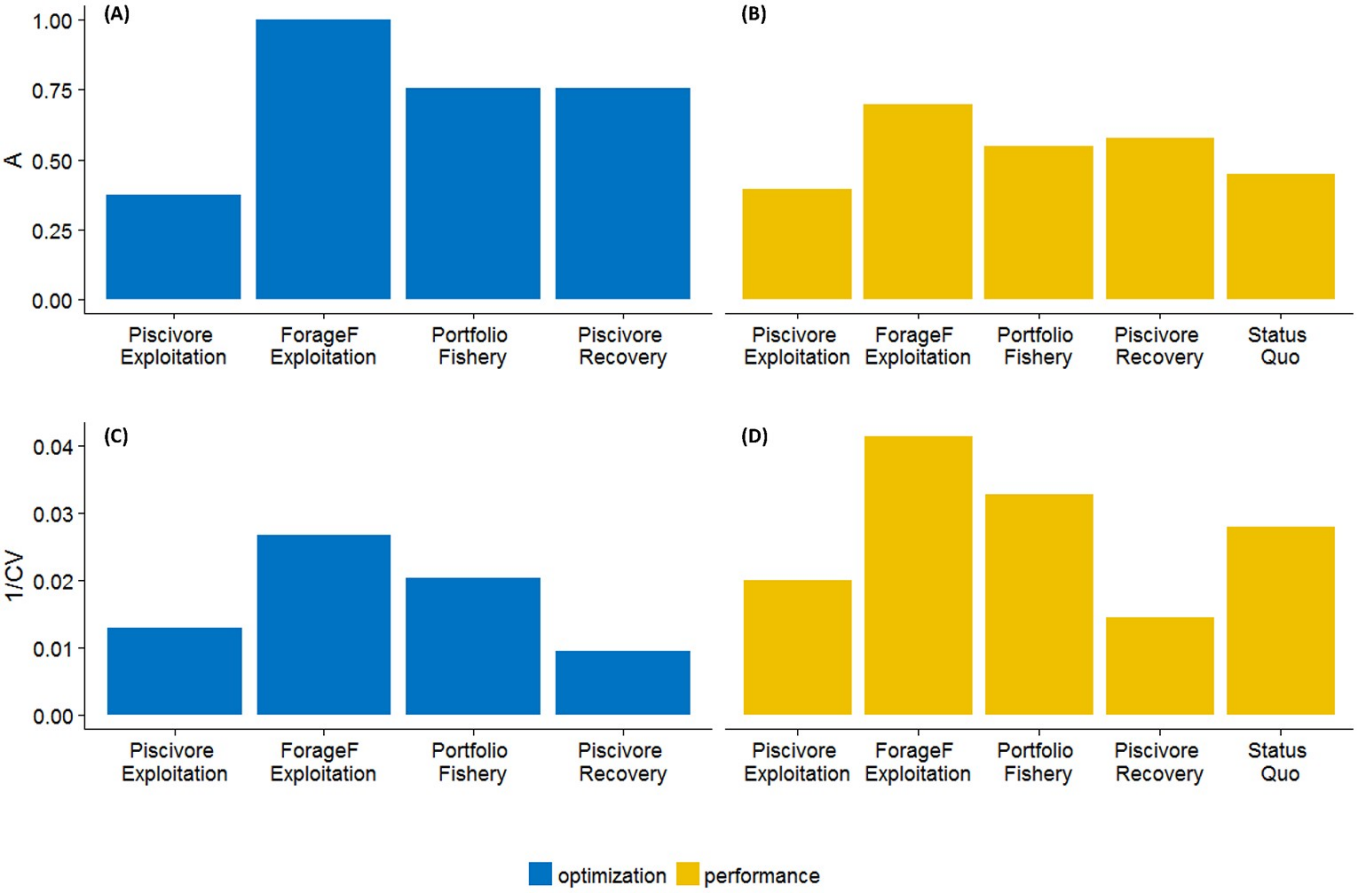
Model agreement measured in two ways in five management scenarios. Model agreement on optimal Fs (A, C, in blue, *i* = cod, herring and sprat) and on performance indicators presented on Fig 6 (B, D, in yellow, *i* = profit bottom trawls, profit pelagic trawls, profit total, SSB cod, SSB herring, SSB sprat). Model agreement was calculated as (A, B) Mean of *A*_*i*_ values (Eq 1) or (C,D) mean of the inverse coefficient of variation values (1*/CV*_*i*_). Fs. Model agreement on Fs cannot be calculated for the ‘Status Quo’ scenario as Fs in this scenario were not estimated but set based on historical values (Table 2).

Model agreement both on Fs maximizing scenario objectives (Fig 7A) and performance indicators (Fig 7B) measured by the *A* index was largest in the ‘Forage Fish Exploitation’ scenario, and lowest in the ‘Piscivore Exploitation’ scenario, with the other two (‘Portfolio Fishery’ and ‘Piscivore Recovery’) scenarios in between. According to *A*, model agreement on the performance of the ‘Status Quo’ scenario was also relatively low.

Compared to *A*, the inverse *CV* (1/*CV*) of model outputs is more focused on the actual values of model outputs instead of their relative trends. Nevertheless, this measurement also highlighted the ‘Forage Fish Exploitation’ scenario as the one with the largest model agreement (Fig 7C and 7D). The biggest difference in model agreement between the two measurements was that 1/*CV* indicated lowest model agreement for the ‘Piscivore Recovery’ scenario instead of the ‘Piscivore Exploitation’ scenario. This has two reasons. First, 1/*CV* overestimates model agreement on ‘Piscivore Expoitation’ as it ignores the Gadget model for this scenario which, in contrast to the other two models, suggests that clupeid Fs do not influence the exploitable biomass of cod. Second, 1/*CV* is more sensitive to the relatively large numeric uncertainty in cod SSB in the ‘Piscivore Recovery’ scenario (Fig 6D).

## Discussion

We use three ecosystem models of the Baltic Sea to study the sensitivity of the F-yield relationship and modelled management scenarios to model uncertainty. Substantial differences among our models in terms of complexity and the processes they include lead to relatively large differences in the F-yield curves (Fig 4). Despite of that, in many cases the models deliver consistent answers both in terms of how to adjust fishing mortality rates to maximize an objective and which scenarios will perform best according to selected indicators. This consistency is highly relevant for management advice. The comparison of model agreement among scenarios suggests that in the Baltic Sea, advice on scenarios aiming to maximize profits of the pelagic fishery (‘Forage Fish Exploitation’) or the total fishery (‘Portfolio Fishery’) is less sensitive to the modelling approaches used than those maximizing profits of the demersal fishery (‘Piscivore Exploitation’) or which have objectives related to a desired fish community composition (‘Piscivore Recovery’). This is understandable as the latter two scenarios are more sensitive to how the feedback from forage fish to cod is represented in the models.

### Model agreement and disagreement on advice regarding fishing mortalities and their consequences

The models gave consistent advice on how to change exploitation levels to achieve certain goals for a subset of the alternative management scenarios. The models agreed that fishing mortalities on herring and sprat could be increased compared to their 2011–2013 values to achieve high profits of the pelagic fishery (‘Forage Fish Exploitation’ scenario) and the total fishery (‘Portfolio Fishery’ scenario). The latter scenario result is also in agreement with the study of [63], whose reported optimal fishing mortality values of sprat and herring to achieve maximum profits of the Baltic fisheries would also represent an increase compared to 2011–2013 values. In the ‘Portfolio Fishery’ scenario, our models provided contradictory advice on the fishing mortality of cod, indicating high uncertainty in the trade-offs between the cod and clupeid fisheries. Overall model uncertainty was smallest for the ‘Forage Fish Exploitation’ scenario, as models mostly agreed that reducing the piscivore predator, cod, is necessary to maximise profits of the fisheries exploiting its prey, sprat and herring. This is consistent with what is expected from multispecies models based on first principles, as it was shown already by [71]. However, [72] showed that the specification of trophic relationships between a predator and its fished prey strongly influence the predator’s biomass response to both its own and its prey’s exploitation rates. In agreement with this result, the ‘Piscivore Exploitation’ scenario was the most sensitive, among the scenarios that we tested to the modelling approach used.

The expected general behavior in a multispecies system is that decreased fishing on prey contributes to maximizing profits of fleets targeting predators [71]. However, there are several instances when this rule does not hold. The first mechanism is the competitive interaction among several prey species or between predator and prey. For example, in the ‘Piscivore Exploitation’ scenario, the EwE model suggests to slightly increase, instead of decrease the F of herring, related to competition between herring and sprat for pelagic food or between herring and cod for benthic food. A second mechanism, not implemented in any of the models presented here, is the predation of prey on predator’s eggs, as is, to some extent, the case in the Baltic Sea [73,74]. If sprat and herring predation on cod eggs was considered, then fishing mortality rates of clupeids in the ‘Piscivore Exploitation’ scenario would need to ensure enough food for cod, while keeping predation on cod eggs low. Third, growth rate of the predator may not be impacted by the availability of forage fish, e.g. if alternative food sources are present [75]. In this case the exploitation level of forage fish is not influential on predator yields and profits, as implied by the Gadget model here.

The example of the ‘Piscivore Exploitation’ scenario shows that analysing model advice for extreme management scenarios before using them to evaluate and advise on more realistic and complex scenarios, could help in identifying the sources of major differences among models. Conducting such an analysis would also indicate the scenarios for which simpler models could reliably provide ecosystem advice without the need to involve more complex models that are more data-hungry, costly, and time-consuming to run and validate.

### Effects of trophic interactions and the environment on model predictions

The interaction parameters [76] and different representation of environmental influences [10] are likely the most important model components causing deviations among model predictions. Although seal predation on fish stocks is represented both in EwE and Gadget, impacts of the seal population are much more pronounced in Gadget. This is probably related to thelinear positive function between and prey mortality in Gadget, while in EwE prey mortality saturates at high seal biomass (Table 1).

Accounting only for top-down effects of predators on prey, but not for bottom-up feedbacks, was considered sufficient by [77] for the classic tactical management of most fish stocks, i.e. to define annual catch quotas. EBFM requires holistic models to highlight long term trade-offs that are relevant for strategic management advice, which may trigger bottom-up effects becoming equally relevant as top-down effects [78]. The evaluated models differed in the strength of bottom-up effects of clupeids on cod. This is partly because the empirical evidence on the importance and direction of those effects at the population level is inconclusive, despite several studies investigating the issue (reviewed by [79]). The clarification of this trophic link would have major implications for cod fishery management scenarios in the central Baltic Sea. In the European context, where the CFP aims at the MSY exploitation of all targeted fish in an ecosystem, we highlight the importance of model choices on the impacts of forage fish on predators. The sensitivity of predatory fish dynamics to prey exploitation levels increases with the strength of the bottom-up effects [80]. Thus, our results suggest that it is necessary to couple ecological theories with empirical efforts to increase knowledge on effects of seal predation on fish stock dynamics and bottom-up effects of prey on predators to be able to provide more robust advice for EBFM.

In terms of abiotic environmental scenarios, EwE and MSPM agreed that nutrient loads have a comparable impact on cod biomass and catches to fishing. This result is supported by the multimodel study of [81] where a strong effect of nutrient management on cod was shown as well. In EwE, nutrient management also strongly affected sprat biomass, mostly via increased primary productivity in the BAU scenario. This is similar to the pattern described by [82] based on historical data. EwE is the only model where the magnitude of abiotic effects is comparable to that of fishing on the biomass dynamics of several stocks. Thus, EwE was the only model where the required change of direction in Fs to achieve management objectives were dependent on environmental scenarios, especially in the ‘Piscivore Recovery’ management scenario. This result highlights the usefulness of complex ecosystem models, including indirect effects of various abiotic environmental pressures, to point out when advice may need to consider such effects.

### Limitations and further steps

The relative simplicity of the Baltic Sea food web was ideal for our study as it enabled us to focus on model formulations, instead of, for example, different definitions of functional groups. However, in other, more diverse, systems the latter can be a major source of model disagreements [42]. In those systems it is especially important to develop more formal protocols to make certain decisions more systematic during model development, i.e. the number and specification of included groups [83].

A typical shortcoming of fisheries-focused ecosystem models is not accounting for economic and especially social drivers of the system [9,20]. This is also true for our models and we acknowledge that our profit estimations can only be interpreted as proxies. Fuel price and market demand are classic examples of important aspects which affect fisheries profits. However, profit estimations here were meant to highlight relative differences among management scenarios, instead of trying to make realistic predictions of their exact outcomes. Relative differences among scenarios are not necessarily highly affected by external factors, such as prices and demand for fish, which are largely driven by the global economy and lifestyles. A possible improvement for real-life usage of the modelling frameworks would be to account for national differences in the profit and costs. Such differences exist, especially in costs, although regarding prices the Baltic market is relatively homogeneous as vessels are free to choose their landing ports. Some dynamic feedbacks between ecosystem and economic components could not be captured using the simplistic approach of the models presented. A larger integration of economics into ecosystem models could be achieved, for example, by treating economic data as any other data component in Gadget, by using an effort- instead of fishing mortality based approach in EwE or by linking different types of modelling approaches together, including ecosystem and economic models and data (e.g. as in [84]). Stakeholders are generally very interested in economic and social outcomes of the management scenarios.

Another problematic issue commonly occurring in long-term simulations is that some parameters affecting growth, predation and reproduction in the ecosystem models are fixed. Thus, the results of our extreme scenario simulations are prone to process error. This is a problem for all models to a varying degree, but it is difficult to estimate to what extent. This issue may be investigated in the future, for example by running a Management Strategy Evaluation with different models using Atlantis or similar whole-system models as an operational model [3,12]. A related issue is that we did not include simulations using a range of parameters by each model to estimate parameter uncertainty and we did not examine the models’ sensitivity to input data. Existing methods to conduct such sensitivity analyses are highly model-specific and the comparability of the resulting parameter uncertainty ranges is questionable. In addition, complete sensitivity analysis may be impossible in case of some models such as EwE and Gadget [26,76]. This is an argument to use a number of complementary modelling approaches to provide advice, as model uncertainty tends to be larger than parameter uncertainty.

When we quantified overall model agreement, we considered all models as equally valid representations of the ecosystem. We are aware that this is a simplification and that more sophisticated approaches for model averaging exist, e.g. Bayesian weighting to combine model results based on how well they represent certain processes according to expert opinion, or based on their hindcast ability [22,85,86]. However, the first method is not objective, and the second method has a limitation in that hindcast ability does not necessarily reflect forecast ability and it is sensitive to errors in data [5]. We argue that model weighting is less of a problem here as our model ensemble is not biased towards a particular method. Nevertheless, to use our models in an actual decision support process, decision support tools that can integrate information from multiple models in relatively flexible ways are useful [87].

## Conclusions

The results showcase the necessity of careful consideration when interpreting ecosystem models to inform management. An *a priori* selection of one model, without an understanding of its associated biases and limitations, may result in misleading conclusions. Therefore, comparison of multiple modelling approaches, as done by the climate change research community, using several Global Climate Models to generate future scenarios [67,88,89] and conservation biologists using multiple Species Distribution Models to support conservation planning [90], is necessary. There are several large-scale fisheries model intercomparison studies underway (e.g. [91]), mostly focusing on climate change impacts on marine ecosystems, often applying a limited range of fisheries forcing. Based on our results, we suggest that it is useful to compare model behavior under a large range of fishing pressures on each stock and their combinations.

EBFM is lagging in implementation partly because of institutional inertia [92], that is, institutions have historically been adjusted to provide advice that seeks to reach narrowly defined targets, such as maximum yield. When various alternative scenarios are presented, it may encourage out-of-the-box thinking, which is important for ecosystem-based scenario planning. Multimodel simulations of alternative scenarios can help to indicate which of those scenarios are robust to the choice of modelling strategy used.

## Supporting information

S1 Appendix Description of models and scenarios used in the study. (DOCX)
