# Supplementary material for: Model uncertainty and simulated multispecies fisheries management advice in the Baltic Sea
Source: PLoS One. 2019 Jan 28;14(1):e0211320. doi: 10.1371/journal.pone.0211320 (PMC6349338; doi:10.1371/journal.pone.0211320)
Supplement: S1 Appendix — (DOCX) [file pone.0211320.s001.docx]

Supplementary Material

Model uncertainty on simulated multispecies fisheries management advice in the Baltic Sea

**Barbara Bauer^*^, Mika Rahikainen, Jan Horbowy, Nataliia Kulatska, Bärbel Müller-Karulis, Maciej T. Tomczak and Valerio Bartolino**

*** Correspondence:** barbara.bauer@su.se

Table of Contents

[1 Ecopath with Ecosim 1](#_Toc525289932)

[2 Gadget 3](#_Toc525289933)

[2.1 Seal predation 4](#_Toc525289934)

[3 Multispecies stock-production model 6](#_Toc525289935)

[3.1 Description of the model 6](#_Toc525289936)

[3.2 Parameterization of the model and application to the central Baltic (Subdivision 25-32) 7](#_Toc525289937)

[4 Model calibration 10](#_Toc525289938)

[5 BALTSEM model and scenarios 12](#_Toc525289939)

[6 Calculation of profits 16](#_Toc525289940)

[6.1 Data sources 16](#_Toc525289941)

[6.2 Calculation of yearly profits 17](#_Toc525289942)

[7 Rescaling *B_lim_* reference points 18](#_Toc525289943)

# Ecopath with Ecosim

Ecopath with Ecosim (Walters *et al.*, 1997; Christensen and Walters, 2004) is a software suite to build and analyse food-web models ([www.ecopath.org](http://www.ecopath.org/)). Ecosim is the dynamic extension of Ecopath that allows temporal analysis and parameter estimation by fitting the model to time series. Biomass dynamics in Ecosim is based on the master equation

$\frac{dB_{i}}{dt}=g_{i}\cdot\sum_{j=1}^{n} Q_{ij}-\sum_{k=1}^{n} Q_{ki}-\left( m_{i}+y_{i}+e_{i} \right)\cdot B_{i}$ (1.1)

where *dB_i_/dt* is the growth rate during the time interval *dt* of group *i* in terms of its biomass *B_i_*, *g_i_* is the net growth efficiency (production/consumption ratio), *Q_ij_* is the consumption of *i* on its prey *j*, *m_i_* the non-predation (‘other’) natural mortality rate, *y_i_* is fishing mortality rate, *e_i_* is net emigration rate. The Baltic Ecopath with Ecosim model used in this study is described in more detail in Bauer et al. (2018) and in (ICES, 2016).

It was recalibrated for this study using environmental forcing from the output of the BALTSEM model and only those reference time series listed in Table S4.1. Besides, we applied a few additional model updates. We decreased the adult cod production/biomass parameter according to (Svedäng and Hornborg, 2017) and introduced negative biomass accumulation to maintain mass-balance in the model (Table S1.1). We have modified the juvenile cod diet composition (Table S1.2): the ratio of juvenile sprat was decreased and those of *Saduria* *entomon* and mysids were increased to reflect the fact that the juvenile cod group in the model includes those too small to be sampled in the stomach sampling program. The ratio of sprat in the diet of adult cod was set according to stomach content data, not adjusted according to MSVPA results on sprat mortality as has been done in ICES (2016). Finally, the extent of hypoxic areas was used as forcing on *Saduria* *entomon*, other macrobenthos (Sarvala, 1971) and cod feeding (Eero *et al.*, 2015; Köster *et al.*, 2017) as opposed to mysids only (Table S1.2).

**Table A** List of parameter changes compared to Bauer et al. (2018).

| **Group** | **Parameter** | **New value**  **(*old value*)** |
| --- | --- | --- |
| Juvenile cod | Production/biomass  (=total mortality) | 0.78 (*1.062*) |
| Adult cod | Production/biomass  (=total mortality) | 0.6 (*0.885*) |
|  | Biomass accumulation rate | -0.25 (0) |

**Table B** Changes in diet composition for the current study. Values in brackets and italics are those used in Bauer et al. (2018).

| **Prey \ predator** | **Juvenile cod** | **Adult cod** |
| --- | --- | --- |
| Adult herring | 0.0593 (*0.0594*) | 0.487 (*0.42*) |
| Juvenile sprat | 0.2 (*0.3*) | 0.05 (*0.12*) |
| Adult sprat | 0.05 (*0.126*) | 0.1 (*0.278*) |
| *Saduria entomon* | 0.179 (*0.119*) | 0.205 (*0.051*) |
| Other macrobenthos | 0.0254 (*0.00937*) | 0.0013 |
| Mysids | 0.2705 (*0.171*) | 0.067 (*0.0397*) |

**References**

Bauer, B., Meier, H. E. M., Casini, M., Hoff, A., Margonski, P., Orio, A., Saraiva, S., Stenbeek, J., Tomczak, M. T. 2018. Reducing eutrophication increases spatial extent of communities supporting commercial fisheries: a model case study. ICES Journal of Marine Science. (*doi:* [10.1093/icesjms/fsy003](http://dx.doi.org/10.1093/icesjms/fsy003))

Christensen, V., and Walters, C. J. 2004. Ecopath with Ecosim: Methods, capabilities and limitations. Ecological Modelling, 172: 109–139.

Eero, M., Hjelm, J., Behrens, J., Buchmann, K., Cardinale, M., Casini, M., Gasyukov, P., *et al.* 2015. Eastern Baltic cod in distress: Biological changes and challenges for stock assessment. ICES Journal of Marine Science, 72: 2180–2186.

Gordon, C., Cooper, C., Senior, S. A., Banks, H., Gregory, J. M., Johns, T. C., Mitchell, J. F. B., *et al.* 2000. The simulation of SST, sea ice extent and ocean heat transports in a version of the Hadley Centre coupled model without flux adjustments. Climate Dynamics, 16: 147–166.

ICES. 2016. Report of the Working Group on Multispecies Assessment Methods (WGSAM), 10-14 October 2016, Reykjavik, Iceland. ICES CM 2016/SSGEPI:21.: 94 pp.

Köster, F. W., Huwer, B., Hinrichsen, H. H., Neumann, V., Makarchouk, A., Eero, M., Dewitz, B. V., *et al.* 2017. Eastern Baltic cod recruitment revisited - Dynamics and impacting factors. ICES Journal of Marine Science, 74: 3–19.

Quaas, M. F., Froese, R., Herwartz, H., Requate, T., Schmidt, J. O., and Voss, R. 2012. Fishing industry borrows from natural capital at high shadow interest rates. Ecological Economics, 82: 45–52. Elsevier B.V.

Sarvala, J. 1971. Ecology of Harmothoe sarsi (Malmgren) (Polychaeta, Polynoidae) in the northern Baltic area. Ann. Zool. Fenn., 8: 231–309.

Svedäng, H., and Hornborg, S. 2017. Historic changes in length distributions of three Baltic cod (Gadus morhua) stocks: Evidence of growth retardation. Ecology and Evolution, 7: 6089–6102.

Tomczak, M. T., Niiranen, S., Hjerne, O., and Blenckner, T. 2012. Ecosystem flow dynamics in the Baltic Proper-Using a multi-trophic dataset as a basis for food-web modelling. Ecological Modelling, 230: 123–147. Elsevier B.V.

Voss, R., Quaas, M. F., Schmidt, J. O., and Hoffmann, J. 2014. Regional trade-offs from multi-species maximum sustainable yield (MMSY) management options. Marine Ecology Progress Series, 498: 1–12.

Walters, C. J., Christensen, V., and Pauly, D. 1997. Structuring dynamic models of exploited ecosystems from trophic mass-balance assessments. Reviews in Fish Biology and Fisheries, 7: 139–172.

# Gadget

Gadget ("Globally applicable Area Disaggregated General Ecosystem Toolbox") is a statistical model for marine ecosystems (Begley, 2005). Gadget is an age-length structured forward-simulation modelling framework and is characterised as a dynamic minimum realistic model (Plaganyi, 2007). Processes, such as growth, maturation, predation, etc., are generally modelled as dependent on length, but age is also tracked in the models, and data can be compared on either a length and/or age scale (Begley, 2005). Gadget models can be fitted to multiple data sets, with various and sometimes even conflicting information, using weighted likelihood functions. The current model is implemented in R using the package Rgadget (Elvarsson, 2015).

Gadget multi-species models are generally implemented from single species models which are later linked by trophic interactions and/or technical interactions among fisheries targeting more than one species. In this case, we started with the implementation of single species Gadget models of Baltic sprat, central Baltic herring and eastern Baltic cod. The three stocks are built for the same time period (1974-2013) and around a similar quarterly based conceptual model with fishing and natural mortality occurring in all the time steps, recruitment once a year in a specified quarter and one or more surveys occurring in different times of the year. The stocks were then linked into a multi-species model via cod predation (Fig. S2.1).

The model used in this study is derived from the model described in Kulatska et al. (Table S2.2). The main difference is that here we include seal that predates on herring, sprat and cod.


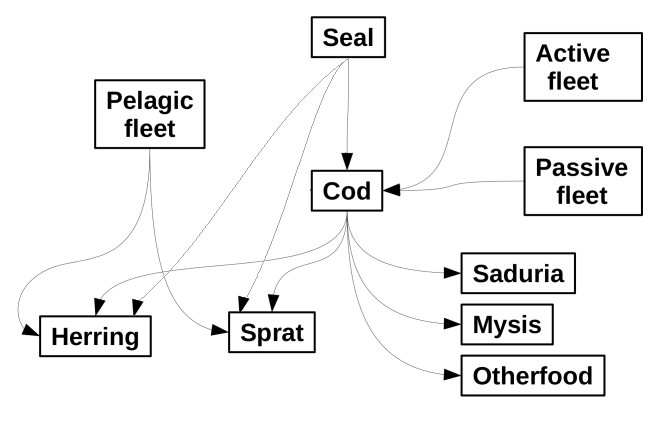


**Figure A** Schematic of Gadget multi-species model.

## Seal predation

Seals in the model act as a fleet. We assumed that individual seal consumes 5 kg of food per day (Lundström, pers. comm.). Cod was assumed to comprise 8% of prey biomass consumed by seal, herring 49% and sprat 11%. Suitability of prey lengths (*l*) for seal predation was modelled with a gamma function as follows:

$$S\left( l \right)=\left( \frac{l}{\alpha\beta} \right)^{\alpha}e^{\left( \alpha- \frac{l}{\beta} \right)}$$

**Table C** List of parameter changes compare to Kulatska et al. (*submitted*)

| **Parameter** | **Cod** | **Sprat** | **Herring** |
| --- | --- | --- | --- |
| p1 in cod-sprat or cod-herring suitability function |  | 1.45 | 1.1 |
| p2 in cod-sprat or cod-herring suitability function, q1 and 2 |  | 0.9295 | 0.3375 |
| p2 in cod-sprat or cod-herring suitability function, q3 and 4 |  | 0.4574 | 0.2103 |
| p3 in cod-sprat or cod-herring suitability function |  | 0.03 | 0.07 |
| p4 in cod-sprat or cod-herring suitability function |  | 0.18 | 0.3 |
| p0 in cod-other food suitability function, q1 and 2 | 0.2158 |  |  |
| p0 in cod-other food suitability function, q3 and 4 | 0.3087 |  |  |
| p0 in cod-saduria suitability function^1^ | 0.4 |  |  |
| p1 in cod-saduria suitability function^1^ | 0 |  |  |
| p2 in cod-saduria suitability function, q1 and 2^1^ | 1.2524 |  |  |
| p2 in cod-saduria suitability function, q3 and 4^1^ | 0.1 |  |  |
| p3 in cod-saduria suitability function^1^ | 0.4 |  |  |
| p0 in cod-mysids suitability function | 1.3 |  |  |
| p2 in cod-mysids suitability function, q1 and 2 | 1.1501 |  |  |
| p2 in cod-mysids suitability function, q3 and 4 | 0.6177 |  |  |
| p3 in cod-mysids suitability function | 1 |  |  |
| recruitment, 1974 |  | 17.1213 | 1.5110 |
| recruitment, 1975 |  | 40.3679 | 2.0093 |
| recruitment, 1976 |  | 13.9619 | 1.8313 |
| recruitment, 1977 |  | 4.2150 | 2.0720 |
| recruitment, 1978 |  | 8.2996 | 1.5977 |
| recruitment, 1979 |  | 6.2085 | 2.5054 |
| recruitment, 1980 |  | 11.6869 | 3.3466 |
| recruitment, 1981 |  | 2.9031 | 2.5979 |
| recruitment, 1982 |  | 10.9412 | 2.0682 |
| recruitment, 1983 |  | 4.6139 | 2.7183 |
| recruitment, 1984 | 5.9647 | 3.0011 | 2.4816 |
| recruitment, 1985 |  | 1.1215 | 1.4368 |
| recruitment, 1986 |  | 3.4061 | 3.1626 |
| recruitment, 1987 | 2.3367 | 1.2551 | 1.0458 |
| recruitment, 1988 | 2.2655 | 3.0143 | 1.8492 |
| recruitment, 1989 | 1.4767 | 3.3156 | 2.1503 |
| recruitment, 1990 | 2.7026 | 3.1304 | 1.7560 |
| recruitment, 1991 | 34.8373 | 6.0602 | 2.3296 |
| recruitment, 1992 | 27.0752 | 8.5779 | 1.8619 |
| recruitment, 1993 | 25.4153 | 4.6710 | 1.4438 |
| recruitment, 1994 | 23.4461 | 11.7774 | 2.2413 |
| recruitment, 1995 | 16.1153 | 8.3456 | 1.8043 |
| recruitment, 1996 | 18.9771 | 1.2455 | 0.9616 |
| recruitment, 1997 | 18.5958 | 8.9579 | 1.6420 |
| recruitment, 1998 | 22.6385 | 0.3435 | 0.8329 |
| recruitment, 1999 | 16.9713 | 6.8432 | 1.7136 |
| recruitment, 2000 | 27.4822 | 2.7404 | 1.4785 |
| recruitment, 2001 | 21.6278 | 3.3863 | 1.3351 |
| recruitment, 2002 | 15.0642 | 6.6717 | 2.2276 |
| recruitment, 2003 | 34.9447 | 12.7020 | 1.3669 |
| recruitment, 2004 | 28.8037 | 2.6835 | 0.8221 |
| recruitment, 2005 | 26.6041 | 6.0551 | 1.5506 |
| recruitment, 2006 | 29.4221 | 9.3516 | 1.3482 |
| recruitment, 2007 | 23.6985 | 4.6422 | 2.5798 |
| recruitment, 2008 | 25.9589 | 10.5029 | 1.8353 |
| recruitment, 2009 | 19.7417 | 2.4441 | 1.6243 |
| recruitment, 2010 | 13.2458 | 4.3670 | 0.8783 |
| recruitment, 2011 | 11.9128 | 7.1779 | 2.7503 |
| recruitment, 2012 | 12.8064 | 7.8929 | 2.9846 |
| recruitment, 2013 | 5.7314 | 6.1769 | 1.3710 |
| α in seal-cod (herring or sprat) suitability function^2^ | 9.4520 | 88.2610 | 24.2151 |
| β in seal-cod (herring or sprat) suitability function^2^ | 6 | 0.1334 | 0.6652 |
| k in sprat growth function |  | 75 |  |
| beta-binomial for sprat growth |  | 1.81 |  |
| α in pelagic trawl fleet suitabilty |  | 0.72 |  |
| l50 in pelagic trawl fleet suitabilty |  | 14.29 |  |
| α in BIAS suitabilty |  | 1.12 |  |
| l50 in BIAS suitabilty |  | 9.13 |  |

^1^ different suitability function was used in Kulatska et al (*submitted*), ^2^ parameters unique to the model used in this study.

**References**

Begley, J. 2005. Gadget user manual. Technical Report 120, Marine Research Institute, Reykjavik.

Elvarsson, B. T. 2015. RGadget: a R–package for development, testing and analysis of Gadget models. ICES C. 2015/A37 2–4.

N. Kulatska, S. Neuenfeldt, U. Beier, B. Elvarsson, H. Wennhage, G. Stefansson, V. Bartolino. (*accepted*) Understanding ontogenetic and temporal variability of Eastern Baltic cod diet using a multispecies model and stomach data

Plaganyi, E. E. 2007. Models for an ecosystem approach to fisheries. FAO Fisheries Technical Paper, No. 477. Rome, Italy.

# Multispecies stock-production model

## Description of the model

The multispecies stock-production model (Horbowy 1996, Horbowy 2005) does not require historical age structure, which is an advantage of the model in data poor situation or when age determination is difficult as in the case of eastern Baltic cod stock (Eero et. al., 2015; Hüssy et. al., 2016). However, to apply the model some age information is needed as the model uses growth parameters which are determined form size at age data.

The model was implemented in components describing adult fish (age 3 and older for cod, and age 2 and older for herring and sprat) and young fish (considered unexploited) (age 1-2 for cod, and age 0-1 for herring and sprat). For adult fish the biomass *B* of stock *s* at the beginning of year *t+1* is

$B_{s}\left( t+1 \right)=B_{s}\left( t \right)e^{a_{s}\left( t \right)}+{Ryoung}_{s}\left( t+1 \right)$ (3.1)

where

$a_{s}\left( t \right)=v_{s}h_{s}w_{s}^{{-1}/3}(t)-k_{s}-q_{s}E_{s}(t)-{M1}_{s}-\sum_{r=1}^{n} h_{r}w_{r}^{-1/3}(t)\frac{G_{r}^{s}B_{r}(t)}{\sum_{j=1}^{n} G_{r}^{j}B_{j}(t)+OT}$ (3.2)

and *Ryoung* is recruitment to adult component (biomass from oldest age in young fish component, which survives unexploited phase); *E* - fishing effort; *q* - catchability coefficient; *M1* - coefficient of natural mortality caused by reasons other than predation (residual natural mortality); *w* - mean weight of fish in the stock; *v, h, k* - growth parameters (fraction of eaten food assimilated for growth, anabolism, and catabolism rates, respectively); *G_r_^s^* - suitability of prey *s* to predator *r*; *OT* - "other food"; *s, r, j* - stocks; *n* - number of stocks. The term after *M1* in expression for *a* (eq. 3.2) represents predation mortality (*M2*).

The dynamic of the young fish component (unexploited part of the stock) is presented by

$N_{si}\left( t+1 \right)=N_{si}\left( t \right)\exp\left[ -{M1}_{s}-\sum_{r=1}^{n} h_{r}w_{r}^{{-1}/3}(t)\frac{G_{r}^{s}B_{r}(t)}{\sum_{j=1}^{n} G_{r}^{j}B_{j}(t)+OT} \right]+R_{s}(t+1)$ (3.3)

$B_{si}(t)=N_{si}(t)w_{si}(t)$ (3.4)

where *N_si_* – stock number at age *i* in stock *s*, *R* is the biomass of the year-class recruited to the stock, *w_si_* – mean weight at age *i* in stock *s*. Recruitment to the unexploited component of the stock was implemented as recruitment estimated in ICES analytical assessment scaled by parameter *u*.

## Parameterization of the model and application to the central Baltic (Subdivision 25-32)

The model was applied to simulate the stock dynamics and trophic interactions of cod, herring, and sprat stocks in the central and eastern Baltic (Sub-divisions 25-32) in 1982-2013. Simulated trophic levels are presented in Fig. S3.1. Adult cod eats everything in the system, young cod eats young herring and sprat and adult sprat. The fishery is on top of the system exploiting adult cod, herring, and sprat.

In classical production models fish growth is assumed constant. In the Baltic, growth of three main species (cod, herring, and sprat) has shown marked declines during three recent decades; as a consequence the weight at age of these species in recent years has been about 40 – 60% lower than in 1980s or beginning of 1990s (Fig. S3.2). It is necessary to model such changes to adequately reproduce stocks dynamics. It appeared that growth of cod may be presented as related to area of hypoxic waters, while herring growth depends on salinity (Fig. S3.2). In case of sprat density dependence in growth has been demonstrated (Fig. S3.2, Horbowy and Swinder, 1989; Casini et al, 2011). Therefore, the sub-models of cod, herring, and sprat growth were developed and included into this version of the multispecies stock-production model. The above dependencies were implemented by making catabolism rate, h:

- linearly related to area of hypoxic waters for cod,
- linearly related to salinity for herring,
- stock-density dependent for sprat (hyperbolical relationship).

This was done basing on relation between asymptotic weight and growth parameters from the present model *Winf=(vh/k)^3^*

Fishing mortality and recruitment estimates from ICES assessment (ICES, 2013) were taken as fishing effort and recruitment indices, respectively. Such approach represents situation when relatively good quality data on fishing effort and recruitment are available.

The unknown parameters of the model (*G, q, u, B0*) were estimated by minimization of the sum of squared differences between observed and modeled values, i.e.

$SS(G,q,u,B0)=\sum_{s,t} \lambda_{Y}(\ln Y_{st}-\ln\underline{Y}_{st})^{2}+\sum_{s,t} \lambda_{sc}(\ln SC_{st}-\ln\underline{SC}_{st})^{2}+\lambda_{\mathbf{B}}(\ln B0_{s}-\ln\underline{B0}_{s})^{2}$ (4)

where *Y* and *Y*, *SC* and *SC*, and *B0* and *B0* denote observed and model catches, stomach contents, and initial biomass, respectively. Index *s* refers to species (cod, herring, sprat) and *t* is year (1982-2013). The parameters λ represent statistical weights which were the inverse of the variance associated with successive residual terms. The parameters *G* are determined relative to a constant multiplier, so the highest was allotted 1, and other *G* values were estimated relative to that. The other food (*OT*) was assumed constant at 1000 units. The model was validated through inspecting the distribution of residuals and retrospective analysis.


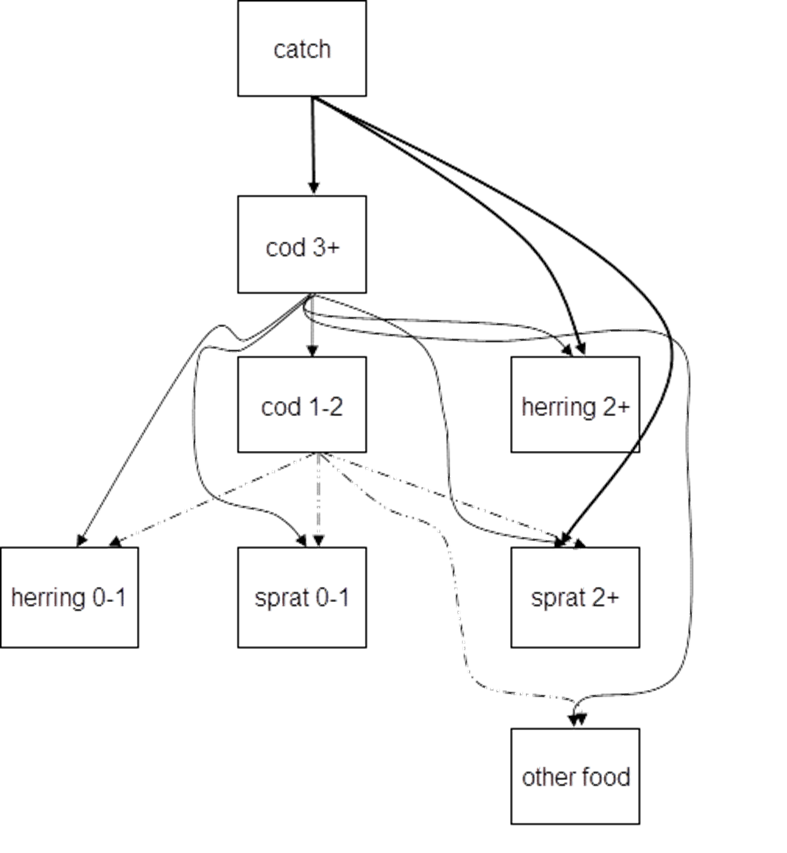


**Figure B** Trophic interactions in the Baltic simulated by the multispecies stock-production model.


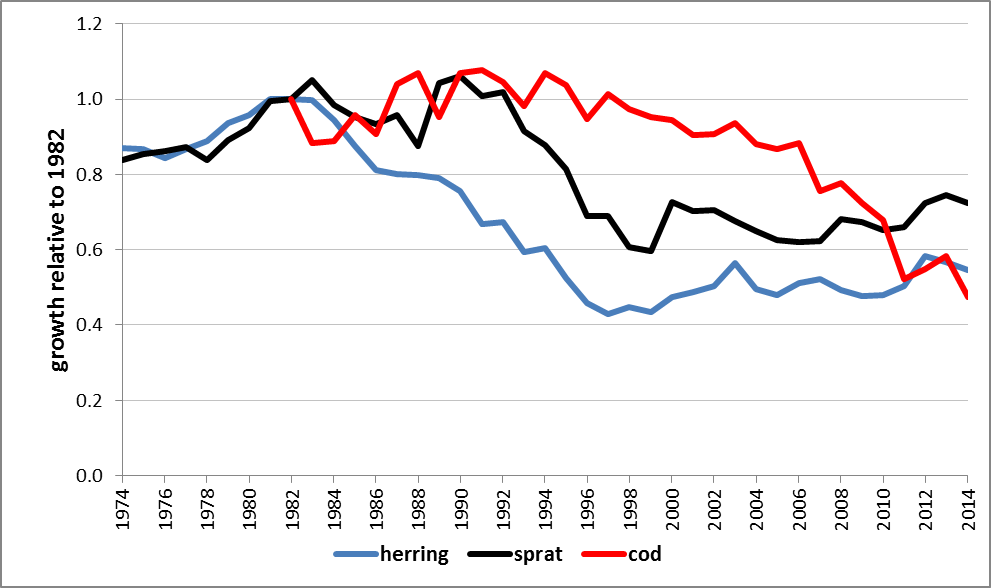


**Figure C** Growth of Baltic cod, herring, and sprat in relative terms (1982=1)

**References**

Christensen, V., and Walters, C. J. 2004. Ecopath with Ecosim: Methods, capabilities and limitations. Ecological Modelling, 172: 109–139.

Eero, M., Hjelm, J., Behrens, J., Buchmann, K., Cardinale, M., Casini, M., Gasyukov, P., *et al.* 2015. Eastern Baltic cod in distress: Biological changes and challenges for stock assessment. ICES Journal of Marine Science, 72: 2180–2186.

Gordon, C., Cooper, C., Senior, S. A., Banks, H., Gregory, J. M., Johns, T. C., Mitchell, J. F. B., *et al.* 2000. The simulation of SST, sea ice extent and ocean heat transports in a version of the Hadley Centre coupled model without flux adjustments. Climate Dynamics, 16: 147–166.

ICES. 2016. Report of the Working Group on Multispecies Assessment Methods (WGSAM), 10-14 October 2016, Reykjavik, Iceland. ICES CM 2016/SSGEPI:21.: 94 pp.

Köster, F. W., Huwer, B., Hinrichsen, H. H., Neumann, V., Makarchouk, A., Eero, M., Dewitz, B. V., *et al.* 2017. Eastern Baltic cod recruitment revisited - Dynamics and impacting factors. ICES Journal of Marine Science, 74: 3–19.

Quaas, M. F., Froese, R., Herwartz, H., Requate, T., Schmidt, J. O., and Voss, R. 2012. Fishing industry borrows from natural capital at high shadow interest rates. Ecological Economics, 82: 45–52. Elsevier B.V.

Sarvala, J. 1971. Ecology of Harmothoe sarsi (Malmgren) (Polychaeta, Polynoidae) in the northern Baltic area. Ann. Zool. Fenn., 8: 231–309.

Svedäng, H., and Hornborg, S. 2017. Historic changes in length distributions of three Baltic cod (Gadus morhua) stocks: Evidence of growth retardation. Ecology and Evolution, 7: 6089–6102.

Tomczak, M. T., Niiranen, S., Hjerne, O., and Blenckner, T. 2012. Ecosystem flow dynamics in the Baltic Proper-Using a multi-trophic dataset as a basis for food-web modelling. Ecological Modelling, 230: 123–147. Elsevier B.V.

Voss, R., Quaas, M. F., Schmidt, J. O., and Hoffmann, J. 2014. Regional trade-offs from multi-species maximum sustainable yield (MMSY) management options. Marine Ecology Progress Series, 498: 1–12.

Walters, C. J., Christensen, V., and Pauly, D. 1997. Structuring dynamic models of exploited ecosystems from trophic mass-balance assessments. Reviews in Fish Biology and Fisheries, 7: 139–172.

# Model calibration

The three models used in the study were calibrated using the same datasets as far as possible. For this purpose, a common database was set up and queried using R functions from the package ‘mfdb’ (Lentin 2014). The database contained cod stomach data from (ICES, 2016b) which served as the basis of cod diet composition in all three models. We distinguish between ‘reference time series’ and ‘forcing time series’. ‘Reference time series’ (in Gadget terminology ‘likelihood components’) are those time series not needed for forward projections, but provide information to estimate model parameters during the model fitting procedure. ‘Forcing time series’ represent external factors affecting the system and need to be specified for forward projections. Reference and forcing time series relating to cod, sprat and herring used by all three models to generate hindcasts are listed in Table D1. Reference time series relating to other groups, which is only relevant for EwE, are listed in ICES (2016a). During forward projections, a range of fishing mortality values were applied (see main text ‘Optimal fisheries strategy search’) as forcing in all three models, together with seal biomass (EwE, Gadget) and/or environmental (EwE, MSPM) forcing. The latter is described in the following section 5.

**Table D** Reference and forcing time series. Environmental forcing time series are described in section 5 ‘BALTSEM model and scenarios’. BITS: Baltic International Trawl Survey, WGBFAS: ICES Working Group for Baltic Sea Fisheries Assessment, STECF: European Commission, Scientific, Technical and Economic Committee for Fisheries, BIAS: Baltic International Acoustic Survey.

| **Group** | **Component** | **Source** | **Time series type** | **Model** | **Used as** |
| --- | --- | --- | --- | --- | --- |
| Cod | Stock abundance | BITS | Biomass of adult and juvenile age groups | EwE | reference |
|  | Stock abundance | BITS | Indices of abundance in numbers by length groups | Gadget | reference |
|  | Stock age-size structure | BITS | Length and age-length distributions | Gadget | reference |
|  | Formulation of growth submodels | WGBFAS | Weight at age in the stock | MSPM | reference |
|  | Fisheries catch amount | WGBFAS* | Catch of adults in biomass (EwE: also juveniles), Gadget total catch per fleet in biomass 2004-2013 | EwE, MSPM, Gadget | reference |
|  | Fisheries catch amount | WGBFAS* | Total catch in biomass per fleet 1974-2003 | Gadget | forcing |
|  | Catch age-size structure | WGBFAS* | Stock structure in catch (age 1974-1999, length 2000-2013) | Gadget | reference |
|  | Fishing effort | STECF | Fishing effort 2004-2013 | EwE, Gadget | forcing |
|  | Fishing mortality | WGBFAS assessments | Fishing mortality | MSPM | forcing |
|  | Indices of recruitment | WGBFAS assessments | Recruitment | MSPM | forcing |
| Herring, sprat | Stock abundance | BIAS | Biomass per stanza | EwE | reference |
|  | Stock abundance | BIAS | Individual and aggregated age groups. | Gadget | reference |
|  | Stock age-size structure | BIAS | stock structure in survey (length) | Gadget | reference |
|  | Catch amount | WGBFAS* | Catch of adults in biomass (EwE: also juveniles) | EwE, MSPM | reference |
|  | Catch amount | WGBFAS* | Total catch per stock | Gadget | forcing |
|  | Catch age-size structure | WGBFAS* | Stock structure in catch | Gadget | reference |
|  | Formulation of growth submodels | WGBFAS | Weight at age in the stock | MSPM | reference |
|  | Fishing mortality | WGBFAS assessments | Fishing mortalities (yield/biomass) | EwE, MSPM | forcing |

* originally from national landings statistics and data collection

**References**

Christensen, V., and Walters, C. J. 2004. Ecopath with Ecosim: Methods, capabilities and limitations. Ecological Modelling, 172: 109–139.

Eero, M., Hjelm, J., Behrens, J., Buchmann, K., Cardinale, M., Casini, M., Gasyukov, P., *et al.* 2015. Eastern Baltic cod in distress: Biological changes and challenges for stock assessment. ICES Journal of Marine Science, 72: 2180–2186.

Gordon, C., Cooper, C., Senior, S. A., Banks, H., Gregory, J. M., Johns, T. C., Mitchell, J. F. B., *et al.* 2000. The simulation of SST, sea ice extent and ocean heat transports in a version of the Hadley Centre coupled model without flux adjustments. Climate Dynamics, 16: 147–166.

ICES. 2016. Report of the Working Group on Multispecies Assessment Methods (WGSAM), 10-14 October 2016, Reykjavik, Iceland. ICES CM 2016/SSGEPI:21.: 94 pp.

Köster, F. W., Huwer, B., Hinrichsen, H. H., Neumann, V., Makarchouk, A., Eero, M., Dewitz, B. V., *et al.* 2017. Eastern Baltic cod recruitment revisited - Dynamics and impacting factors. ICES Journal of Marine Science, 74: 3–19.

Quaas, M. F., Froese, R., Herwartz, H., Requate, T., Schmidt, J. O., and Voss, R. 2012. Fishing industry borrows from natural capital at high shadow interest rates. Ecological Economics, 82: 45–52. Elsevier B.V.

Sarvala, J. 1971. Ecology of Harmothoe sarsi (Malmgren) (Polychaeta, Polynoidae) in the northern Baltic area. Ann. Zool. Fenn., 8: 231–309.

Svedäng, H., and Hornborg, S. 2017. Historic changes in length distributions of three Baltic cod (Gadus morhua) stocks: Evidence of growth retardation. Ecology and Evolution, 7: 6089–6102.

Tomczak, M. T., Niiranen, S., Hjerne, O., and Blenckner, T. 2012. Ecosystem flow dynamics in the Baltic Proper-Using a multi-trophic dataset as a basis for food-web modelling. Ecological Modelling, 230: 123–147. Elsevier B.V.

Voss, R., Quaas, M. F., Schmidt, J. O., and Hoffmann, J. 2014. Regional trade-offs from multi-species maximum sustainable yield (MMSY) management options. Marine Ecology Progress Series, 498: 1–12.

Walters, C. J., Christensen, V., and Pauly, D. 1997. Structuring dynamic models of exploited ecosystems from trophic mass-balance assessments. Reviews in Fish Biology and Fisheries, 7: 139–172.

# BALTSEM model and scenarios

BALTSEM is a biogeochemical model of the entire Baltic Sea that treats its model domain as a system of thirteen horizontally homogeneous subbasins that are modelled with high vertical resolution. This setup resolves inflow dynamics and stratification in the central deep basins while it keeps computation time low. This approach allows extensive model calibration and validation and quick scenario runs. Gustafsson (2003) give a detailed description of the hydrodynamic module in BALTSEM. The biogeochemical module consists of an NPZD-model (**n**utrient-**p**hytoplankton-**z**ooplankton-**d**etritus) coupled to a simple sediment module. Together they simulate the turnover of nitrogen, phosphorus, and dissolved silica coupled to oxygen dynamics. Three groups of phytoplankton (diatoms, flagellates and nitrogen-fixing cyanobacteria) assimilate dissolved inorganic nutrients according to Redfield ratios. Phytoplankton biomass is grazed by a generalized “heterotroph” organism group and all non-living organic matter is collected by detritus state variables. Sinking detritus transports nitrogen, phosphorus and silica to the bottom sediments. Nutrient release from sediments depends on oxygen concentrations in the bottom water. Oxygen dynamics are in turn simulated by coupling all autotroph and heterotroph processes to oxygen consumption or production. A detailed description of the biogeochemical processes included in BALTSEM is given in Savchuk (2002) and Savchuk et al. (2012).

BALTSEM was calibrated against nutrient, oxygen and chlorophyll a observations from 1970 – 2006 (Savchuk et al., 2012, Eilola et al., 2011) and validated by a long-term hindcast simulation for 1850-2006 (Gustafsson et al. 2012). BALTSEM has been used in ensemble simulations investigating the impact of climate change and different nutrient load scenarios on the Baltic Sea (e.g. Meier et al. 2012 a,b ).

Scenario forcing for foodweb models was generated by continuing a hindcast simulation forced by reconstructed past meteorological conditions and nutrient loads for 1970-2013 into the future, assuming warming according to the ICCP A1B scenario (Nakicenovic et al. 2000). The meteorological forcing is based on dynamically downscaled global climate simulations by the regional model RCAO (Döscher et al. 2002, Meier et al. 2011). We used downscaled runs from the global climate model *HadCM3* (Gordon *et al.*, 2000). Nutrient inputs in the simulations correspond to maximum allowable inputs according to the Baltic Sea Action Plan in its 2013 revision (BSAP; HELCOM 2013); and an increasing loads scenario, where all countries around the Baltic Sea intensified of agriculture production to the high livestock densities of Denmark.

**Table E** BALTSEM forcing implemented in models.

| **Forcing series** | **Group(s)** | **Target variable** | **Model** | **Source (BALTSEM)** |
| --- | --- | --- | --- | --- |
| Cod reproductive volume | cod | egg production rate | EwE | Cod reproductive volume, average May-August |
| Summer sea surface temperature | herring, sprat | egg prodcution rate | EwE | Average T values August (Gadget, sprat: July-August) in SDs 25-29, excl. Gulf of Riga, in depths 0-10 m |
| Salinity | herring | growth rate | MSPM | Surface salinity, annual average, SD 25 |
| Hypoxic area | cod (only when feeding on benthic groups), all benthic groups except of meiobenthos | search rate | EwE | Total hypoxic area in Gotland Sea and Bornholm Basin, yearly average |
|  | cod | growth rate | MSPM |  |
| Spring upper water temperature | *Acartia* spp., *Temora* spp. | search rate | EwE | Average T values March-May of Gotland Sea and Bornholm Basin in depths 10-40 m |
| Primary production forcing | phytoplankton | asymptote of growth equation | EwE | Area-weighted average yearly P/B values, SD 25-29, excl. Gulf of Riga |


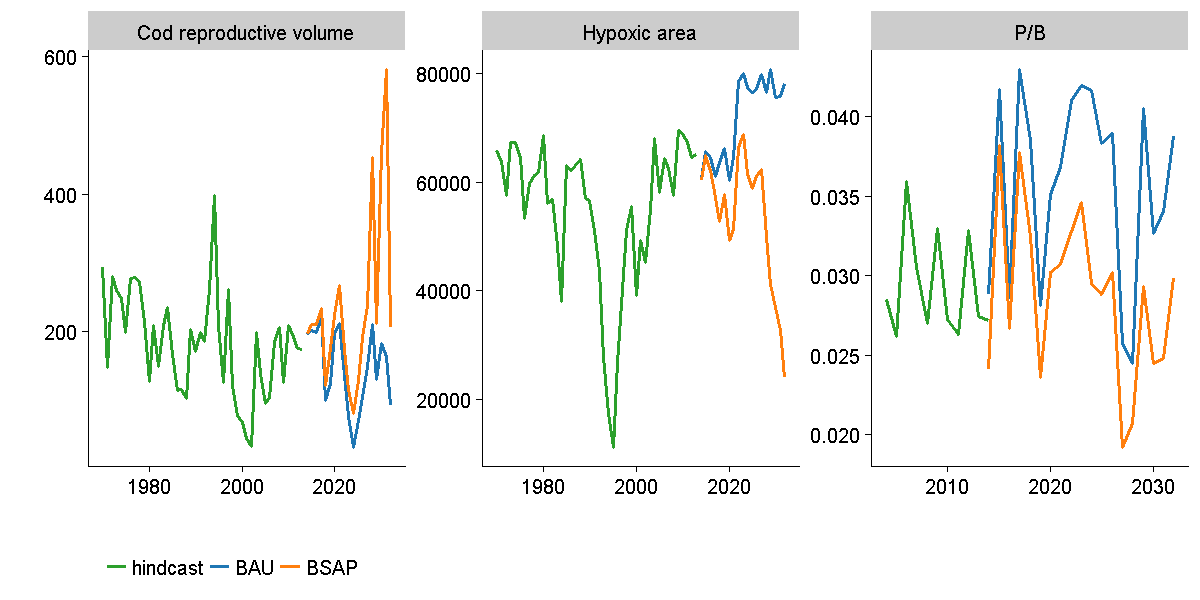


Figure D Temporal forcing of cod reproductive volume (km^3^, left panel), the size of hypoxic areas (km^2^, middle panel) and primary production (production/biomass rate, right panel) calculated from BALTSEM outputs in the historical period (green), Business-As-Usual Scenario (BAU, blue) and Baltic Sea Action Plan Scenario (BSAP, orange). As primary production forcing is only relevant for the EwE model, only values after 2003 are shown.


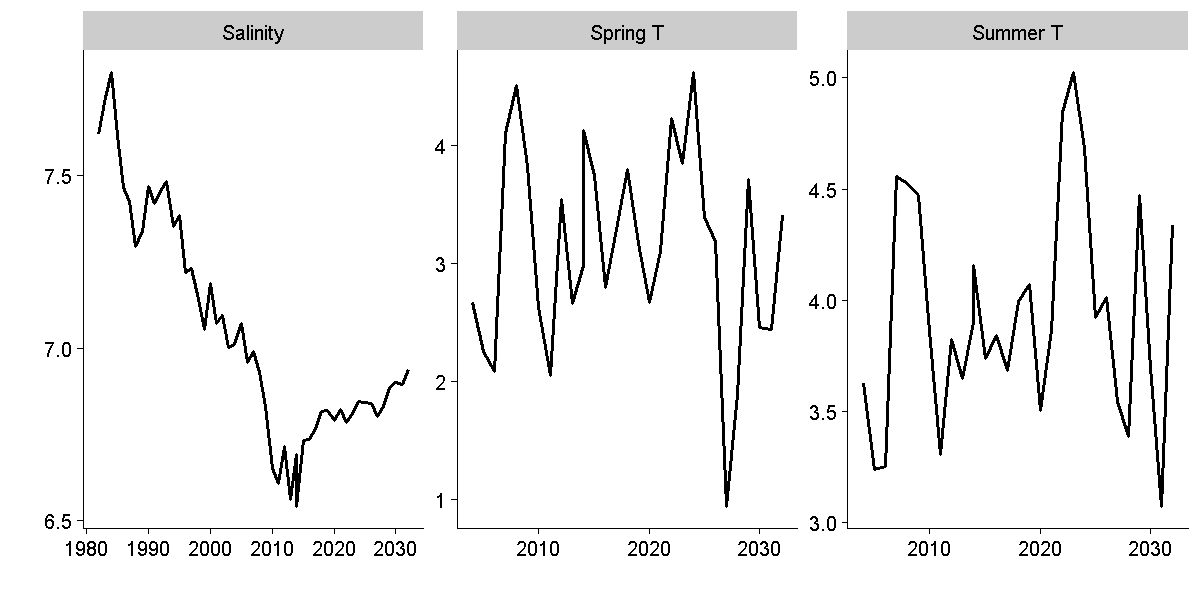


Figure E Temporal forcing of salinity (left panel), spring temperature (˚C, middle panel) and summer temperature (˚C, right panel) calculated from BALTSEM outputs used in the historical period and for all forecasts. As temperature forcing is only relevant for the EwE model, only values after 2003 are shown.

**References**

Döscher, R., Willén, U., Jones, C., & Rutgersson, A. (2002). The development of the regional coupled ocean-atmosphere model RCAO. *Boreal Environment Research*, *7*, 183–912.

Eilola, K., Gustafsson, B. G., Kuznetsov, I., Meier, H. E. M., Neumann, T., & Savchuk, O. P. (2011). Evaluation of biogeochemical cycles in an ensemble of three state-of-the-art numerical models of the Baltic Sea. *Journal of Marine Systems*, *88*(2), 267–284. http://doi.org/10.1016/j.jmarsys.2011.05.004

Gustafsson, B. G., Schenk, F., Blenckner, T., Eilola, K., Meier, H. E. M., Muller-Karulis, B., Neuman, T., Ruoho-Airola, T., Savchuk, O.P., Zorita, E. (2012). Reconstructing the Development of Baltic Sea Eutrophication 1850–2006. *Ambio*, *41*(6), 534–548. <http://doi.org/10.1007/s13280-012-0318-x>

HELCOM. *‎HELCOM Copenhagen Ministerial Declaration.* Taking Further Action to Implement the Baltic Sea Action Plan - Reaching Good Environmental Status for a healthy Baltic Sea. 3 October 2013, Copenhagen, Denmark. <http://helcom.fi/Documents/Ministerial2013/Ministerial%20declaration/2013%20Copenhagen%20Ministerial%20Declaration%20w%20cover.pdf>

Humborg, C., Mörth, C. M., Sundbom, M., & Wulff, F. (2007). Riverine transport of biogenic elements to the Baltic Sea - past and possible future perspectives. *Hydrology and Earth System Sciences Discussions*, *11*(5), 1593–1607.

Meier, M., Höglund, A., Doscher, R., Andersson, H., Loptien, U., & Kjellström, E. (2011). Quality assessment of atmospheric surface fields over the Baltic Sea from an ensemble of regional climate model simulations with respect to ocean dynamics. *Oceanologia*, *53*(1-TI), 193–227.

Meier, H. E. M., Muller-Karulis, B., Andersson, H. C., Dieterich, C., Eilola, K., Gustafsson, B. G., Höglund, A., Hordoir, R., Kuznetsov, I., Neumann, T., Ranjbar, Z., Savchuk, O.P., Schimanke, S. (2012). Impact of Climate Change on Ecological Quality Indicators and Biogeochemical Fluxes in the Baltic Sea: A Multi-Model Ensemble Study. *Ambio*, *41*(6), 558–573. <http://doi.org/10.1007/s13280-012-0320-3>

Meier, H. E. M., Hordoir, R., Andersson, H. C., Dieterich, C., Eilola, K., Gustafsson, B. G., Höglund, A., Schimanke, S. (2012). Modeling the combined impact of changing climate and changing nutrient loads on the Baltic Sea environment in an ensemble of transient simulations for 1961–2099. *Climate Dynamics*. <http://doi.org/10.1007/s00382-012-1339-7>

Nakicenovic, N., J. Alcamo, G. Davis, B. de Vries, J. Fenhann, S. Gaffin, K. Gregory, A. Grubler, Jung, T.Y., Kram, T., La Rovere, E.L., Michaelis, L., Mori, S., Morita, T., Pepper, W., Pitcher, H., Price, L., Riahi, K., Roehrl, A., Rogner, H., Sankovski, A., Schlesinger, M., Shukla, P., Smith, S., Swart, R., van Rooijen, S., Victor, N., Dadi, Z. 2000. Emission scenarios. A special report of working group III of the intergovernmental panel on climate change, Cambridge University Press, 599 pp.

Savchuk, O. P. (2002). Nutrient biogeochemical cycles in the Gulf of Riga: scaling up field studies with a mathematical model. *Journal of Marine Systems*, *32*(4), 253–280

# Calculation of profits

## Data sources

Prices of each species (cod, herring, sprat) were based on data from Swedish sales notes, average values 2011-2015, which were similar to average prices of fish landed in Poland by EU fishers during the same time period. Thus, we considered them representative of Baltic prices.

Based on the work by Quaas *et al.* (2012) and Voss *et al.* (2014) we assume costs in the cod fishery to depend on fishing mortality caused by the fishery, and in schooling fisheries (clupeids) on landed weight. To calculate these coefficients, we used total costs reported for the relevant fleet segments by STECF (yearly average from 2008-2010), divided by F, instantaneous fishing mortality (we used F estimates from the Gadget model hindcast for cod). Cost data was only available at the level of the FAO area 27 (NorthEast Atlantic), but for our calculation we needed to estimate costs at the level of the central Baltic Sea area (ICES Subdivisions 25-29), for all fleet segments separately. We assumed that the ratio of costs of a fleet segment within the central Baltic to the total costs in the whole FAO 27 area is the same as its ratio of landings. We used cost data from the table ‘2016-07_STECF_EU Fleet Economic data_fs level’ and landings from ‘2016-07_STECF_EU Fleet Landings FAO Gear levels’ (both downloaded from *stecf.jrc.ec.europa.eu/reports*) to rescale costs to the central Baltic Sea. On ‘total costs’, here we mean the sum of the following reported cost categories: ‘Crew costs’, ‘Unpaid labor costs’, ‘Energy costs’, ‘Repair costs’, ‘Other variable costs’ (i.e. those related to fishing effort). We related our three fleet segments to STECF gear categories in the following way: BT (‘bottom trawlers’) comprise DRB (dredgers), DTS (demersal trawlers/seiners), MGO (vessels using other active gears), MGP (vessels using polyvalent active gears), PS (purse seiners) and TBB (beam trawlers). GN (‘gillnetters’) comprise DFN (drift/fixed netters), FPO (vessels using pots/traps), HOK (hooks), PG (passive gears), PGO (vessels using other passive gears), PGP (polyvalent passive gears only). PT (‘pelagic trawls’) correspond to TM (pelagic trawlers).

For clupeids we used values directly from Voss *et al.* (2014), who estimated them based on STECF data of pelagic trawler and seiner fleets (2002-2008).

**Table F** Prices and cost parameters used to calculated profits in future scenarios.

| **Species** | **Parameter** | **Value** | **Unit** |
| --- | --- | --- | --- |
| Cod | price | 1.61 | EUR/kg |
|  | cost coefficient (BT) | 7.2 x 10^7^ | EUR/F |
|  | cost coefficient (GN) | 12 x 10^7^ | EUR/F |
| Herring | price | 0.44 | EUR/kg |
|  | cost coefficient | 0.15 | EUR/kg |
| Sprat | price | 0.2 | EUR/kg |
|  | cost coefficient | 0.11 | EUR/kg |

Besides direct consumption of the target species cod, seals were assumed to impact passive gears through damaging their catches and therefore decreasing their landings in EwE and Gadget. The amount of damaged catch was a linear function of seal abundance. The coefficient (*b*) describing the relationship between seal abundance and damaged catch was derived assuming that the amount of discarded catch equals the landings of the hauls with reported seal-gillnet interactions (SWAM, 2014). The coefficient was thus calculated based on the information from the Swedish logbook for the period 2001-2012 on the proportion of catches affected by seals and estimations of grey seal abundance.

## Calculation of yearly profits

*Profit* is calculated as the earning before interests, taxes depreciation and amortization (EBITDA). For a given year: profit= landings*price – cost

The amount of cod landings for the BT and GN fleet segments were direct model outputs (Gadget) or were calculated from total cod landings (EwE, MSPM) as described in the main text (see subsection ‘Performance indicators’). Prices and costs were estimated as described above. Profits are calculated separately for herring and sprat and summed up to calculate profits of PT.

In the EwE and Gadget models, we deducted a certain proportion of the cod catch damaged by seals from total cod catches of gillnetters in year *t* ($C_{GN, t}$) to calculate their landings in year *t* ($L_{GN, t}$):

$$L_{GN,t}= C_{GN,t}-C_{GN,t} \times(b \times N_{t})$$

where $N_{t}$ is the total number of seals in year *t* and *b* =1.025 x 10^-5^, estimated as described above. In the EwE model seals were simulated as biomass, and were converted to numbers assuming that one seals weighs 100 kilograms (Tomczak et al., 2012).

**References**

Quaas, M. F., R. Froese, H. Herwartz, T. Requate, J. O. Schmidt, and R. Voss. 2012. Fishing industry borrows from natural capital at high shadow interest rates. *Ecological Economics* 82. Elsevier B.V.: 45–52. doi:10.1016/j.ecolecon.2012.08.002.

Swedish Agency for Marine and Water Management. 2014. Sälpopulationernas tillväxt och utbredning samt effekterna av sälskador i fisket. Gothenburg. Retrieved from https://www.havochvatten.se/hav/uppdrag--kontakt/publikationer/publikationer/2015-01-14-salpopulationernas-tillvaxt-och-utbredning-samt-effekterna-av-salskadorna-i-fisket.html

Tomczak, M.T., Niiranen, S., Hjerne, O., Blenckner, T., 2012. Ecosystem flow dynamics in the Baltic Proper-Using a multi-trophic dataset as a basis for food-web modelling. Ecol. Modell. 230, 123–147. doi:10.1016/j.ecolmodel.2011.12.014

Voss, R., M. F. Quaas, J. O. Schmidt, and J. Hoffmann. 2014. Regional trade-offs from multi-species maximum sustainable yield (MMSY) management options. *Marine Ecology Progress Series* 498: 1–12. doi:10.3354/meps10639.

# Rescaling *B_lim_* reference points

*B_lim_* values were taken from ICES assessments (ICES 2016a) and rescaled in each model using the slope and intercept of a linear regression between *SSB*s from model hindcasts (EwE: 2004-2013, Gadget: 1991-2013, MSPM: 1982-2013) and corresponding ICES estimates. E.g. if a model tended to underestimate *SSB*s compared to ICES for a species, the *B_lim_* value for that species and model was lower than the ICES value. In the case of *B_lim,s_* in the EwE model the ratio of average model hindcast *SSB_s_* to average *SSB_s_* by ICES was used for rescaling, as the R^2^ from the linear regression was low (0.32).

**Table G** Original ICES *B_lim_* values (last row) and rescaled values for all models.

| **model** | ***B_lim,c_*** | ***B_lim,h_*** | ***B_lim,s_*** |
| --- | --- | --- | --- |
| EwE | 72 | 560 | 216 |
| Gadget | 58 | 375 | 276 |
| MSPM | 91 | 419 | 484 |
| ICES | 63 | 430 | 410 |
